# The role of commensal microbes in the lifespan of *Drosophila melanogaster*

**DOI:** 10.18632/aging.102073

**Published:** 2019-07-12

**Authors:** Hye-Yeon Lee, Shin-Hae Lee, Ji-Hyeon Lee, Won-Jae Lee, Kyung-Jin Min

**Affiliations:** 1 Department of Biological Sciences, Inha University, Incheon 22212, South Korea; 2 School of Biological Sciences, Seoul National University, Seoul 08826, South Korea

**Keywords:** lifespan, commensal microbe, abundance, composition, *Drosophila melanogaster*

## Abstract

Commensal microbes have mutualistic relationships with their host and mainly live in the host intestine. There are many studies on the relationships between commensal microbes and host physiology. However, there are inconsistent results on the effects of commensal microbes on host lifespan. To clarify this controversy, we generated axenic flies by using two controlled methods – bleaching and antibiotic treatment – and investigated the relationship between the commensal microbes and host lifespan in *Drosophila melanogaster*. The removal of microbes by using bleaching and antibiotic treatments without detrimental effects increased fly lifespan. Furthermore, a strain of flies colonized with a high load of microbiota showed a greater effect on lifespan extension when the microbes were eliminated, suggesting that commensal bacteria abundance may be a critical determinant of host lifespan. Consistent with those observations, microbial flora of aged fly gut significantly decreased axenic fly lifespan via an increase in bacterial load rather than through a change of bacterial composition. Our elaborately controlled experiments showed that the elimination of commensal microbes without detrimental side effects increased fly lifespan, and that bacterial load was a significant determinant of lifespan. Furthermore, our results indicate the presence of a deterministic connection between commensal microbes and host lifespan.

## INTRODUCTION

Commensal microbes with symbiotic relationships with their hosts have been actively investigated in several fields of research in attempts to elucidate their interaction with the host. In particular, many studies have been initiated since the development and wide application of metagenomic sequencing analysis. In recent years, studies on the treatment and prevention of diseases through the production of changes in the host’s intestinal environment have been carried out, and many research teams have suggested that the intestinal microbiota can affect the host’s metabolic diseases [1], immune diseases [2], development [3, 4], reproduction [5], biorhythms [6], and even behavior and mood [7].

Studies into the roles of microbiota as they relate to host aging and lifespan have also been conducted. In *Caenorhabditis elegans* grown axenically, developmental time and lifespan were about twice as long as those in control worms [8]. Furthermore, the use of microorganisms as food items and the secondary metabolites secreted by the commensal microbes are reported to affect the lifespan of *C. elegans* [9, 10]. The viability of axenic animals suggests that the presence of microbes in the gut is neither obligatory nor essential to the host’s development, under appropriate conditions. However, conflicting results have been reported on the effects of commensal microbe presence on the lifespan of *Drosophila melanogaster*. Brummel *et al.* observed that the lifespan of flies reared axenically following egg bleaching or antibiotic treatment was shorter than that of conventionally reared fly, and that effect could be recovered by exposure of the flies to microbes within 2–3 days from eclosion [11]. In contrast, Yamada *et al*. did not show an alteration of lifespan in axenic condition flies, while Clark *et al.*, Petkau *et al.*, Galenza *et al*., Tefit *et al*. and Obata *et al.* showed that the absence of commensal microbes extends the lifespan in *Drosophila* [14–19]. Ren *et al.*, Ridley *et al.*, and Iatsenko *et al*. showed increased lifespan of *Drosophila* following egg bleaching or antibiotic treatment, but the increases did not reach statistical significance [12, 13, 20]. To clarify the reasons for these inconsistencies, we generated axenic flies using highly elaborate well-controlled methods and observed that elimination of commensal microbes without detrimental side effects increased host lifespan. Moreover, we observed that an age-related increase in microbial load significantly decreased host lifespan, and a change in microbial load had a more critical effect than that from an age-related change in microbial composition.

## RESULTS

### Lifespan of axenic *D. melanogaster* from bleached eggs

**Figure 1:**
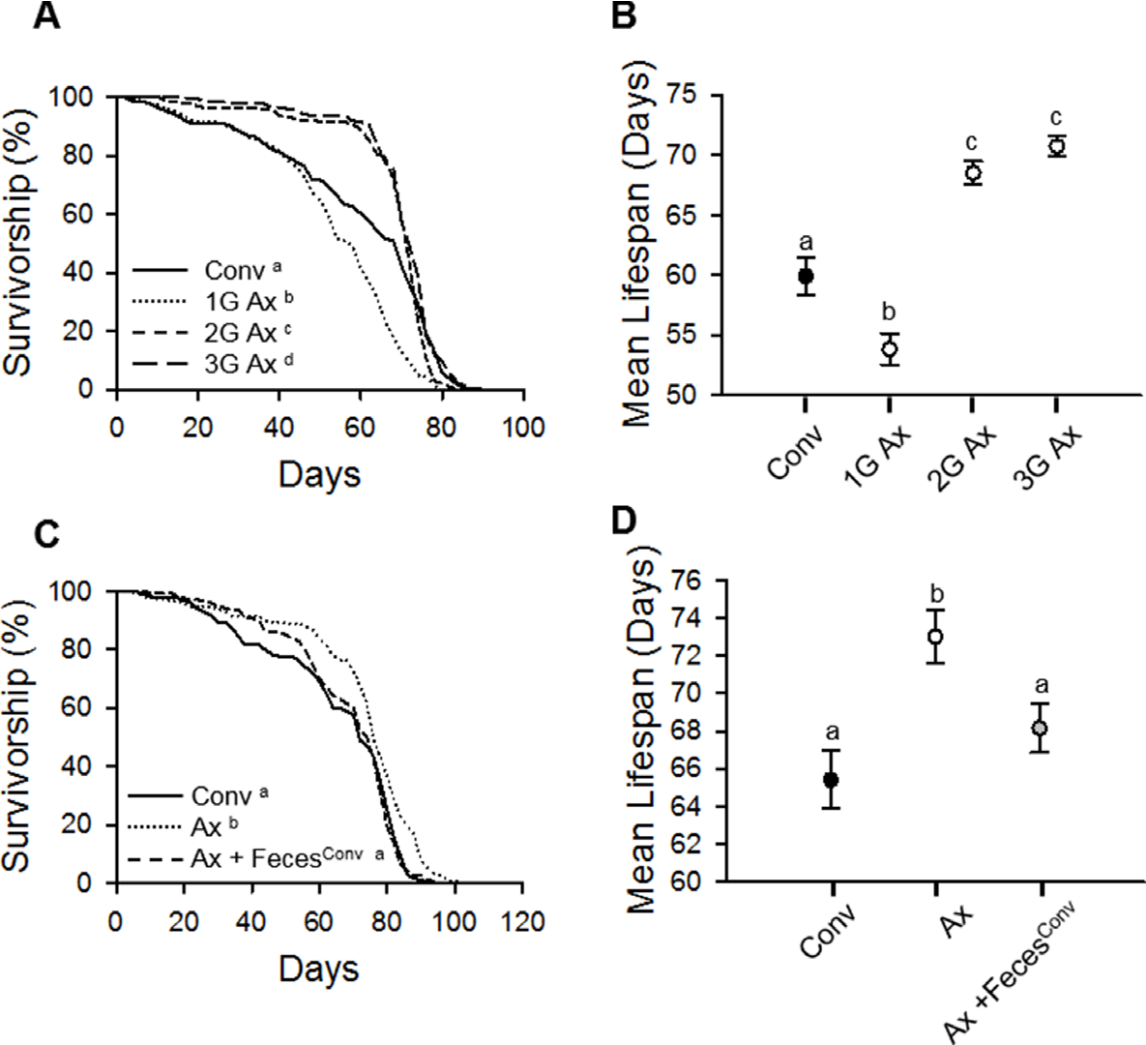
**Elimination of commensal microbes by egg bleaching extends host lifespan.** (**A**) Survival of first (1G), second (2G), and third (3G) generations of Ax flies from bleached eggs. (**B**) Mean lifespan of Conv fly and 1G, 2G, and 3G of Ax flies from bleached eggs. Filled dot, Conv flies; open dots, Ax flies. (**C**) Survival of Ax flies treated with feces from 10-day-old Conv flies. (**D**) Mean lifespan of Ax flies treated with feces from 10-day-old Conv flies. Black dot, Conv flies; white dot, Ax flies fed feces from Ax flies; gray dot, Ax flies fed feces from Conv (Ax + Feces^Conv^) flies. Different letters indicate significant differences between groups. Error bars represent the SEM.

To clarify the effect of an absence of commensal microbes on host lifespan, we generated axenic (Ax) flies of laboratory wild-type strain *w^1118^ D. melanogaster* by using a sodium hypochlorite-based bleaching method as previously reported [15]. The mean lifespan of the first generation (1G) Ax flies hatched from bleached eggs was shorter than that of conventionally reared (Conv) flies (Figure 1A and 1B, Conv fly 59.92 ± 1.52 days; 1G Ax fly 53.83 ± 1.31 days, 10.16% decrease, log-rank test, χ^2^ = 38.85, *p* < 0.0001, Wilcoxon test, χ^2^ = 20.81, *p* < 0.0001). Because the bleaching process might have a deleterious effect on the health of flies, we measured the lifespan of second- (2G) and third- (3G) generation flies after first-generation bleaching. Interestingly, the 2G and 3G Ax flies had increased lifespans compared to that of Conv flies (Figure 1A and 1B, Supplementary Table 1; 2G Ax fly 68.52 ± 0.94 days, 14.35% increase, log-rank test, χ^2^ = 0.08, *p* = 0.77, Wilcoxon test, χ^2^ = 8.91, *p* < 0.005; 3G Ax fly 70.73 ± 0.85 days, 18.04% increase, log-rank test, χ^2^ = 8.20, *p* < 0.005, Wilcoxon test, χ^2^ = 18.93, *p* < 0.0001), suggesting that the bleaching method may have a detrimental effect on *D. melanogaster*, but the effect diminished over subsequent generations. To confirm whether the lifespan extension by egg bleaching is due to the absence of commensal microbes, we introduced microbes from 10-day-old Conv flies to 3-day-old 3G Ax flies by using fecal transplantation. As expected, the longevity effect of bacterial removal in Ax flies was diminished by fecal microbe transplantation (Figure 1C and 1D, Supplementary Table 1; Ax 73.00 ± 1.40 days; Ax + Feces^Conv^ 68.15 ± 1.30 days, 6.64% crease, log-rank test, χ^2^ = 22.61, *p* < 0.0001). These results indicate that the bleaching of eggs can have an adverse effect on adult fly lifespan, but the elimination of commensal microbes increases the lifespan of subsequent generations of flies.

### Lifespan of axenic *D. melanogaster* generated by antibiotic treatment

**Figure 2:**
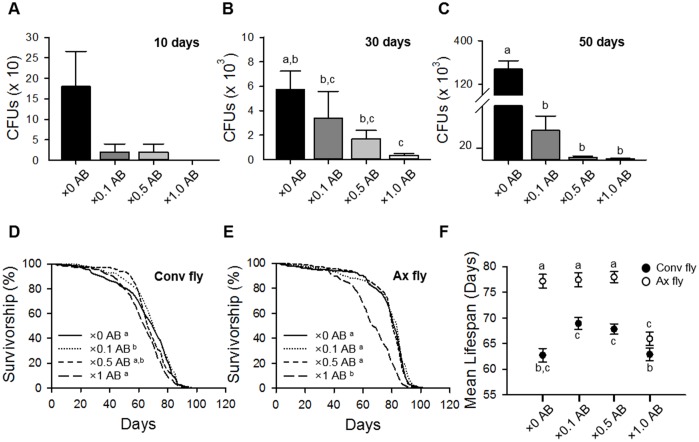
**Elimination of commensal microbe by antibiotic treatment extends host lifespan.** (**A**–**C**) Colony-forming units (CFUs) of Conv flies treated with ×0.1, ×0.5, or ×1 of the antibiotic cocktail AB for 10 days (**A**), 30 days (**B**), and 50 days (**C**). (**D**–**E**) Survival of flies treated with ×0.1, ×0.5, or ×1 AB in Conv fly (**D**) and Ax fly (**E**). (**F**) Mean lifespan of flies treated with ×0.1, ×0.5, or ×1 AB. Filled dots, Conv flies; open dots, Ax flies. Different letters indicate significant differences between groups. Error bars represent the SEM.

To confirm the effect of microbe removal on host lifespan, we supplied an antibiotic (AB) cocktail to Conv flies as described previously [12]. In our laboratory, bacteria were colonized into the guts of 10-day-old control flies at densities of up to 1.8 × 10^2^ cells per fly; colonization was increased with fly age to densities of up to 2.2 × 10^5^ cells per fly in 50-day-old flies (Figure 2A–2C). Treatment with the AB cocktail reduced the number of bacteria in the gut, and the AB effects were gradually decreased by dilution of the cocktail (Figure *2*A–2C). Following AB treatment (×1 AB), the mean lifespans of Conv flies were not significantly different from that of untreated (×0 AB) Conv flies (Figure 2D and 2F, Supplementary Table 1; Conv + ×0 AB 62.73 ± 1.32 days; Conv + ×1.0 AB 62.89 ± 1.21 days, log-rank test, χ^2^ = 0.53, *p* = 0.47). However, AB cocktail treatment dramatically reduced the lifespan of Ax flies at the ×1 AB concentration (Figure 2E and 2F, Supplementary Table 1; Ax + ×0 AB 77.17 ± 1.32 days; Ax + ×1.0 AB 65.94 ± 1.34 days, 14.55% decrease, log-rank test, χ^2^ = 64.01, *p* < 0.0001). We assumed that the AB cocktail concentration used might have had a toxic effect on fly health. To investigate this possibility, we treated flies with the AB cocktail at 2- and 10-fold concentration dilutions (×0.5 AB and ×0.1 AB, respectively). The ×0.5 AB and ×0.1 AB cocktail treatments did not significantly affect the lifespan of Ax flies (Figure 2E and 2F, Supplementary Table 1; Ax + ×0.1 AB 77.44 ± 1.34 days, log-rank test, χ^2^ = 1.41, *p* = 0.23; Ax + ×0.5 AB 77.97 ± 1.06 days, log-rank test, χ^2^ = 0.64, *p* = 0.42), whereas the lifespan of Conv flies was increased at these lower AB concentrations (Figure 2D and 2F, Supplementary Table 1; Conv + ×0.1 AB 68.93 ± 1.16 days, 9.88% increase, log-rank test, χ^2^ = 12.33, *p* < 0.001; Conv + ×0.5 AB 67.84 ± 0.99 days, log-rank test, χ^2^ = 2.86, *p* = 0.09). These results indicate that an AB treatment at a dosage that did not have toxic effects can increase the lifespan of *D. melanogaster*, which is consistent with the results obtained by using the bleaching method to produce Ax flies.

### Strain-specific longevity effect of commensal microbe elimination

**Figure 3:**
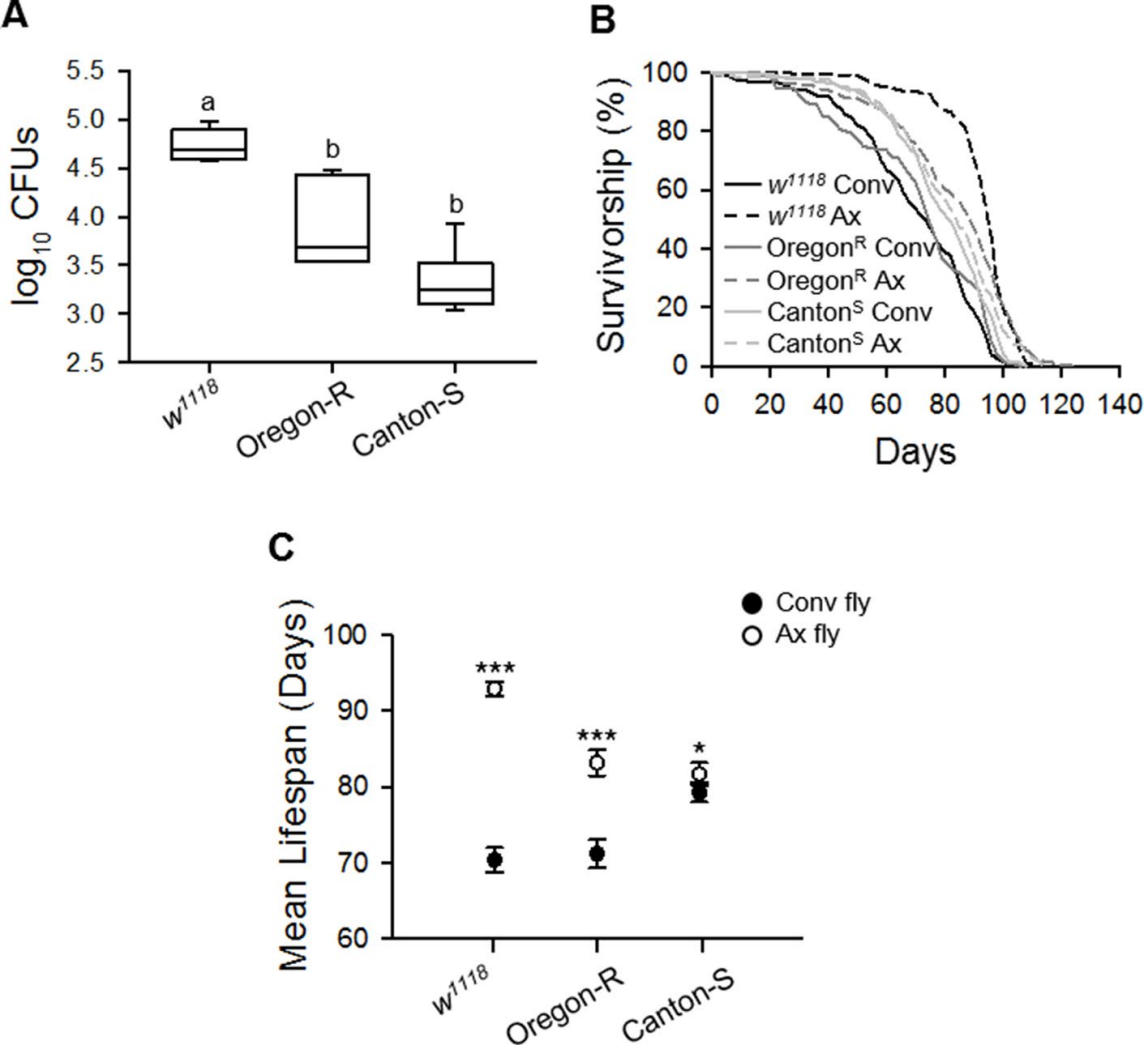
**Lifespan extension effect of the elimination of commensal microbes differs among laboratory fly strains.** (**A**) The total number of CFUs from 1-week-old flies in plate count agar (PCA) media. (**B**) Survival curve of laboratory wild-type *w^1118^*, Oregon-R, and Canton-S strains with the elimination of microbes. Solid lines, Conv flies; dashed lines, Ax flies. (**C**) Mean lifespan of laboratory wild-type *w^1118^*, Oregon-R, and Canton-S strains following the elimination of microbes. Filled dots, Conv flies; open dots, Ax flies. Asterisks indicate significant differences between Conv flies and Ax flies (log-rank test, *p < 0.05, ***p < 0.0001). Error bars represent the SEM.

Han *et al*. (2017) reported that *Drosophila* with different genetic backgrounds had different microbial flora, even though the flies were reared under the same conditions in the same laboratory [21]. Since a different microbial flora may lead to different lifespan results following bacterial removal, we compared the lifespan of Ax flies generated from different strains in our laboratory; wild-type Oregon-R, Canton-S, and *w^1118^* strains. Under the same conditions, the load levels of commensal microbes residing in these strains were different; the number of colony-forming units (CFUs) of commensal microbes was lowest in the Canton-S strain and highest in the *w^1118^* (Figure 3A, ANOVA, *p* < 0.0001; Tukey’s HSD test, *w^1118^ vs.*OR, *p* < 0.0001; *w^1118^ vs.*CS, *p* < 0.0001; OR *vs.*CS, *p* < 0.005). In all three strains, the bleached egg effect on the longevity of 3G Ax flies was observed, but the extent of lifespan extension resulting from bacterial removal was different among the strains. Bacterial removal increased the lifespan of *w^1118^* flies by 32.01%, whereas the lifespan increases were only 16.82% and 3.03% in the Oregon-R and Canton-S strains, respectively (Figure 3B and 3C, Supplementary Table 1, log-rank test, *w^1118^*, χ^2^ = 146.33, *p* < 0.0001; Oregon-R, χ^2^ = 37.43, *p* < 0.0001; Canton-S, χ^2^ = 6.60, *p* < 0.05). Moreover, the extent of the lifespan extension was positively correlated with the abundance of commensal microbes residing in each strain (Spearman’s correlation, rho = 0.88, *p* < 0.0001). To confirm that the longevity effect produced by axenic culture was related to bacteria abundance in flies, we performed an analysis of covariance (ANCOVA) test to adjust the bacterial loads among three strain groups. Following the ANCOVA test and after adjustment of bacterial abundance, we confirmed that the differences in the lifespan extension effect of axenic culture among the three strains disappeared (ANCOVA, *p* = 0.99). It showed the possibility that the difference in lifespan extension effect by bacteria removal among these three strains was the result of colonized bacterial abundance.

Taken together, the results showed that the elimination of commensal bacteria using a method that is not accompanied by detrimental side effects can increase the lifespan of flies. In addition, strains colonized with a high level of commensal bacteria exhibit a greater increase in lifespan following bacteria elimination.

### Age-related commensal microbe flora changes shorten the lifespan of axenic *D. melanogaster*

Since the *Drosophila* strain colonized with the higher level of commensal microbes showed a greater effect of bacterial elimination on longevity, we hypothesized that the commensal microbial load can determine host lifespan. To investigate this hypothesis, we supplied homogenates of flies of different ages to Ax flies and determined the effects on lifespan.

The commensal microbe floral abundance is widely reported to increase with host age [12, 15, 22, 23], and our 16S rRNA PCR and CFU results also showed that the abundance of commensal bacteria significantly increases with fly age (Supplementary Figure 1A and 1B). In addition, by using 454-pyrosequencing analysis, we observed that the composition of commensal bacteria also changes with age (Supplementary Figure 1C–1E). In 10-day-old (young) flies, there were 328 operational taxonomic units (OTUs) assigned; while, in 50-day-old (old) flies, 635 OTUs were assigned (CD-HIT 99% threshold) (Supplementary Figure 1C, Supplementary Table 2), indicating that the compositional richness of the microbial species in the gut flora increased with age. At the phylum level, *Proteobacteria* (including *Acetobacter* and *Komagataeibacter*) and *Firmicutes* (including *Lactobacillus* and *Leuconostoc*) comprised 99% of the microbiome in the *D. melanogaster* in this study (Supplementary Table 3)*.* At the species level, *Acetobacter persici* JCM25330(T) (*Ap*, 45.29% of microbiome) and *Lactobacillus brevis* ATCC14869(T) (*Lb*, 34.25%) were dominant in young flies, while *Acetobacter malorum* LMG1746(T) (*Am*, 54.51%) and *Lactobacillus plantarum* ATCC14917(T) (*Lp*, 24.47%) were dominant in old flies (Supplementary Figure 1E, Supplementary Table 3). Moreover, the proportions of *Ap* (5.92%) and *Lb* (1.33%) were markedly reduced in old flies when compared with the levels in young flies. *Komagataeibacter medellinensis* (13.72%) and *Leuconostoc pseudomesenteroides* (11.33%) were detected in young and old flies, respectively (Supplementary Figure 1E, Supplementary Table 3).

**Figure 4:**
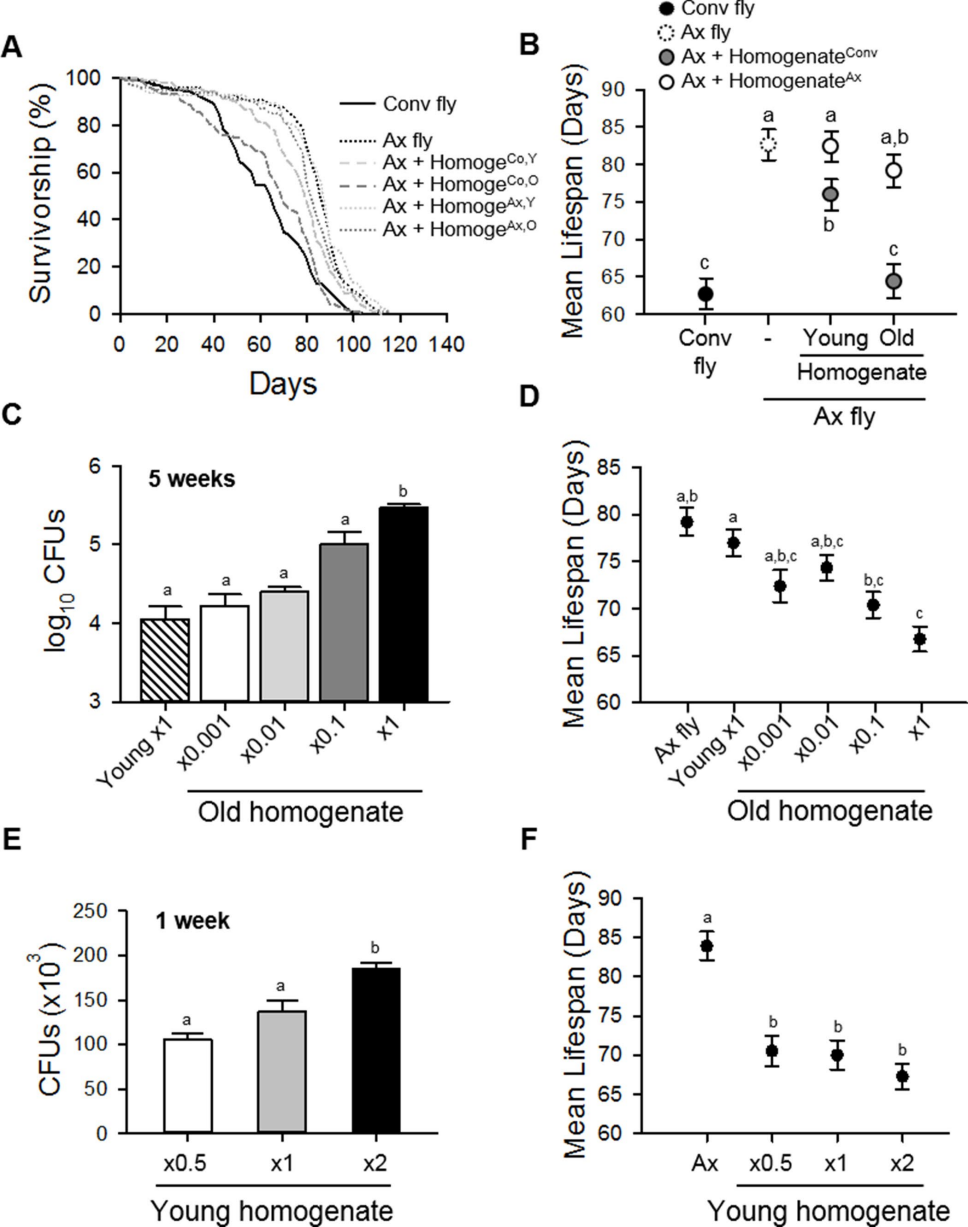
**Homogenate of Conv fly decreases the lifespan of Ax fly.** (**A**) Survival curves of Conv or Ax flies fed fly body homogenates from 10-day-old (young) or 50-day-old (old) flies. Solid lines indicate Conv flies, dotted lines indicate Ax flies or Ax flies fed Ax fly homogenate, and dashed lines indicate Ax flies fed Conv fly homogenate. Homoge^Co,Y^ indicates homogenate from young Conv flies, Homoge^Co,O^ indicates homogenate from old Conv flies, Homoge^Ax,Y^ indicates homogenate from young Ax flies, and Homoge^Ax,O^ indicates homogenate from old Ax flies. (**B**) Mean lifespan of Ax flies fed homogenate of young or old Conv or Ax flies. Black dot, Conv flies; dashed line, Ax flies; gray dots, Ax + Homogenate^Conv^; white dots, Ax + Homogenate^Ax^. (**C**) CFUs of Ax flies fed young fly homogenate or serially diluted old fly homogenate. CFUs were determined 5 weeks after the initial feeding. (**D**) Mean lifespan of Ax flies after feeding homogenates of young or serially diluted old Conv flies. (**E**) CFUs of Ax flies fed young fly homogenate concentrated at ×0.5, ×1, and ×2. CFUs were measured 1 week after the initial feeding. (**F**) Mean lifespan of Ax flies after feeding homogenates of young Conv flies. Homogenate from young Conv flies was diluted or concentrated up to two-fold. Different letters indicate significant differences between groups. Error bars represent the SEM.

To determine whether age-related microbial changes can affect host lifespan, we fed body homogenates from 10-day-old (young) or 50-day-old (old) Conv flies to 3-day-old Ax flies. Consistent with above results, the lifespan of the Ax flies was longer than that of Conv flies (Figure 4A and 4B, Supplementary Table 4; Conv fly 62.74 ± 2.08 days; Ax fly 82.64 ± 2.05 days, 31.72% increase, log-rank test, χ^2^ = 45.98, *p* < 0.0001). When the body homogenate from young flies was fed to Ax flies, the Ax fly lifespan was decreased by 8.12% compared to the lifespan of non-fed Ax flies, while the lifespan of Ax flies fed body homogenate from old flies was decreased by 22% (Figure 4A and 4B, Supplementary Table 4; Ax + Homogenate^Co,Y^ 75.93 ± 2.09 days, log-rank test, χ^2^ = 4.61, *p* < 0.05; Ax + Homogenate^Co,O^ 64.46 ± 2.32 days, log-rank test, χ^2^ = 43.12, *p* < 0.0001). A deleterious effect of body homogenate feeding on Ax fly lifespan was not observed when Ax flies were fed body homogenates of young or old Ax flies (Figure 4A and 4B, Supplementary Table 4; Ax + Homogenate^Ax,Y^ 82.39 ± 2.06 days, log-rank test, χ^2^ = 2.43, *p* = 0.12; Ax + Homogenate^Ax,O^ 79.14 ± 2.21 days, log-rank test, χ^2^ = 0.49, *p* = 0.49). We also observed a deleterious effect of commensal microbes in old flies by feeding them with gut homogenates from young or old Conv flies (Supplementary Figure 2 and Supplementary Table 4). These results indicate that the microbiota present in aged flies has a more adverse effect on lifespan than that present in young flies.

To elucidate whether the detrimental effect of microbiota from aged flies was due to the increased load of commensal microbes in old flies, we fed homogenates from 50-day-old (old) flies at several dilution rates to Ax flies. We observed that the Ax flies fed 10^2^–10^3^ times dilutions of old fly homogenate had a similar abundance of microbes to that of flies fed a 10-day-old (young) homogenate, indicating that the young (×1 Y) homogenate, the ×0.001 old (×0.001 O) homogenate, and the ×0.01 O homogenate contained microbial flora with similar microbial abundances but different compositions (Figure 4C). When the diluted old homogenates were fed to Ax flies, the fly lifespan was similar to that of flies fed with young homogenate, while the lifespan of Ax flies fed undiluted old (×1 O) homogenate was markedly decreased (Figure 4D and Supplementary Figure 3A, Supplementary Table 4; Ax + ×1 Y 76.96 ± 1.42 days; Ax + ×0.001 O 72.37 ± 1.71 days, log-rank test, χ^2^ = 2.30, *p* = 0.13; Ax + ×0.01 O 74.33 ± 1.32 days, 3.42% decrease, log-rank test, χ^2^ = 8.35, *p* < 0.005; Ax + ×0.1 O 70.37 ± 1.38 days, 8.56% decrease, log-rank test, χ^2^ = 20.06, *p* < 0.0001; Ax + ×1 O 66.74 ± 1.34 days, 13.28% decrease, log-rank test, χ^2^ = 55.63 *p* < 0.0001). These results indicate that the age-related increase in bacterial load is a strong determinant of host lifespan. To confirm the importance of bacterial load on host lifespan, the lifespan of Ax flies fed concentrated young homogenate was determined. We found that the level of colonized microbes in Ax flies was increased by feeding with concentrated young homogenates (Figure 4E), and the reduction of lifespan of Ax flies was worsened by feeding with a 2-fold concentrate of young fly homogenate (×2 Y) compared to the lifespans of flies fed ×0.5 Y and ×1 Y homogenates (Figure 4F, and Supplementary Figure 3B, Supplementary Table 4; Ax + ×1 Y 69.98 ± 1.87 days; Ax 83.92 ± 1.82 days, 19.92% increase, log-rank test, χ^2^ = 50.50, *p* < 0.0001; Ax + ×0.5 Y 70.52 ± 1.99 days, log-rank test, χ^2^ = 0.61, *p* = 0.44; Ax + ×2 Y 67.28 ± 1.63 days, 3.86% decrease, log-rank test, χ^2^ = 5.40, *p* < 0.05). Taken together, these results indicate that microbial abundance is a stronger determinant of host lifespan than microbial composition.

### Increased microbial load may be a stronger determinant of host lifespan than age-related changes in microbial composition

**Figure 5:**
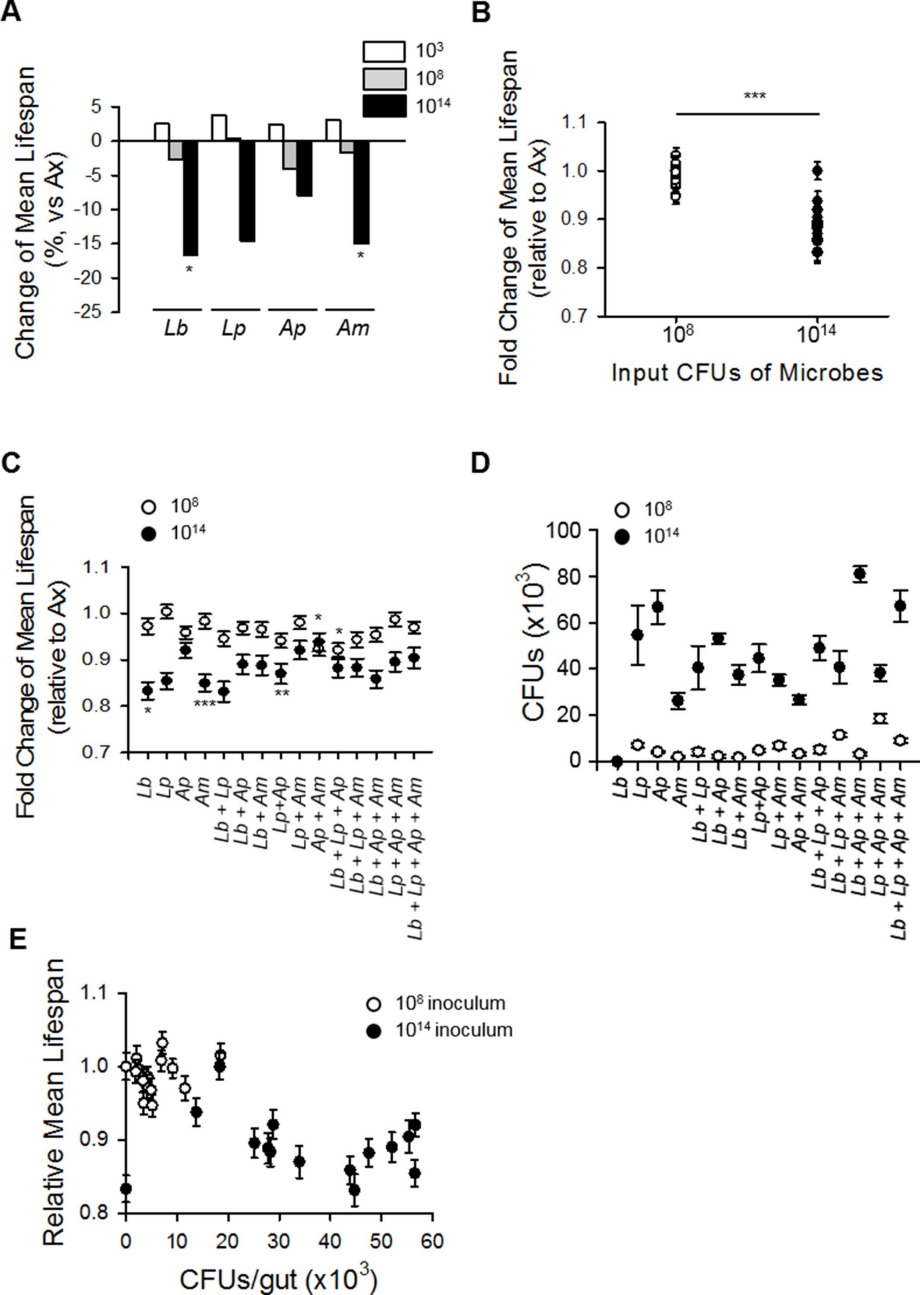
**Increased microbial abundance shortens fly lifespan regardless of microbe composition**. (**A**) Change in mean lifespan of Ax flies inoculated with single species of the dominant microbes at 10^3^, 10^8^, or 10^14^ CFUs. Asterisks indicate significant differences compared to Ax flies. (**B**) Mean lifespan of flies inoculated with combinations of four dominant microbes at 10^8^ or 10^14^ CFUs. Asterisk indicates significant differences between 10^8^ CFUs and 10^14^ CFUs (Spearman’s correlation, rho = −0.79, *p* < 0.0001) (**C**) The mean lifespan of Ax flies inoculated with combinations of four dominant species. Asterisks indicate significant differences compared to Ax flies; all groups with 10^14^ CFUs are significantly reduced compared to Ax flies (log-rank test, *p < 0.05, ***p < 0.0001). (**D**) The CFUs of Ax flies inoculated with combinations of four dominant microbe species. (**E**) Mean lifespan of Ax flies inoculated with combinations of four dominant species as functions of the abundance of the colonized microbe. Abundance of microbes and mean lifespan of flies were negatively correlated (Spearman’s correlation, rho = −0.57, *p* < 0.05). Error bars represent the SEM.

As previously mentioned, both the abundance and composition of commensal microbes change with host age. To further elucidate whether the age-dependent change of microbial abundance is more critical to determining host lifespan than that of microbial composition, we generated gnotobiotic flies inoculated with four of the dominant species of commensal microbes in *Drosophila*: *Lb*, *Lp*, *Ap*, and *Am*. When Ax flies were inoculated with a single species of these microbes (monoxenic) at 10^3^ CFUs, the mean lifespans were increased, but the changes did not reach statistical significance (Figure 5A, Supplementary Figure 4A, ANOVA, *p* = 0.52). In addition, Ax flies mono-inoculated with each bacterium at 10^8^ CFUs lived for a shorter period than the control flies, but those changes also did not have statistical significance (Figure 5A, Supplementary Figure 4B, ANOVA, *p* = 0.18). Interestingly, the inoculated fly's lifespans were decreased with mono-inoculations of each of the four microbes (*Lb*, *Lp*, *Ap*, or *Am*) at 10^14^ CFUs, with the changes in the *Lb* and *Am* groups having statistical significance (Figure 5A, Supplementary Figure 4C, Supplementary Table 5, ANOVA, *p* < 0.0001; Tukey's HSD *vs.* Ax, *Lb*, *p* < 0.005; *Lp*, *p* = 0.06; *Ap*, *p* = 0.08; *Am*, *p* < 0.0001). These results indicate that the effect of commensal bacteria on host lifespan is dependent on the abundance of microbes colonized in the gut and that a high abundance of bacteria decreases the lifespan of flies.

To reveal whether a specific microbial composition can affect host lifespan, we measured the lifespan of Ax flies inoculated with 2^4^ possible combinations of the four dominant commensal microbe species at two concentration levels (10^8^ and 10^14^ CFUs). Similar to the results obtained with the monoxenic flies, the Ax flies inoculated with each microbe combination at 10^8^ CFUs had lifespans similar to that of untreated Ax flies, whereas inoculation of a 10^14^ CFUs microbe combination decreased the inoculated flies mean lifespan (Figure 5B and 5C, 10^8^ CFUs, Ax fly 85.59 days, gnotobiotic flies 82.35 days; 10^14^ CFUs, Ax fly 76.68 days, gnotobiotic flies 67.58 days, 11.87% decrease). In other words, the lifespan of flies inoculated with the higher concentration of bacteria was shorter than that of flies inoculated with the lower concentration of bacteria, indicating that microbial abundance is a strong determinant of lifespan in *Drosophila*.

To verify the effects of microbial abundance on fly lifespan, we measured the actual colonization level of the inoculated bacterial species. Regardless of the inoculated bacterial species composition, the colonized bacterial load was increased to a greater extent with 10^14^ inoculations than with 10^8^ inoculations (Figure 5D). The results indicate the presence of a significant negative correlation between the CFUs in the fly’s body and the lifespan of the fly (Figure 5E, Spearman’s correlation, rho = −0.57, *p* < 0.05).

## DISCUSSION

Commensal microbes in a symbiotic relationship are known to affect host health and aging. In this study, we observed that commensal microbes can affect host lifespan in *D. melanogaster*, and an increase in microbial load in aging flies can be a stronger determinant of lifespan than that from a compositional change in microbiota.

There is a growing body of evidence indicating that commensal microbes, directly and indirectly, affect the lifespan of a host. For example, supplementation with different strains of *Escherichia coli* can affect the lifespan of *C. elegans* through direct, metabolic, or species-specific signals [24]. Also, the lifespan of aged killifish can be improved when they are treated with transplanted feces from young fish [25]. Moreover, germ-free mice have lived longer than their germ-bearing counterparts [26]. Similar to those animal model studies, several studies have shown that commensal microbes have effects on lifespan in *Drosophila*; however, the effects reported have been contradictory.

In this study, we generated axenic flies by using two methods (egg bleaching and antibiotic treatment) and then determined their lifespans. Interestingly, the lifespan of Ax flies was shorter than that of Conv flies after egg bleaching (Figure 1), which indicates that bleaching can have a detrimental effect on lifespan since the effect was diminished as the number of generations of Ax flies increased. In addition, the lifespan of Ax flies was similar to that of Conv flies after antibiotic treatment at a dosage equal to that used by Ren *et al*. (2007) (Figure 2). However, that antibiotic dose appeared to have a toxic effect on Ax flies since dilution of the antibiotic cocktail increased the lifespan of Ax flies, resulting in a longer lifespan in Ax flies than that in Conv flies. Egg bleaching and antibiotic treatment are commonly used methods to generate Ax flies; regardless, our results suggest that the toxic effects of both of those methods should be considered when generating Ax flies. Most authors have not provided information about which fly generation after egg bleaching they used in their studies [11–13, 15, 27–29], and some tested antibiotics within a very narrow concentration window [11, 12, 17, 27, 28, 30] (see Supplementary Table 12). The inconsistencies in the results reported by different groups on the effect of eliminating microbes on host lifespan might be due to differences in the methods used for microbe elimination.

The above-mentioned inconsistencies among results might also be due to the distinct microbial flora within the flies of each laboratory. Flies reared under the same conditions in the same laboratory can have different microbial flora, depending on their genetic background [21]. In this study, we observed that microbial loads were different among the three tested *Drosophila* strains, and the strains colonized with a higher level of commensal bacteria exhibited a greater increase in lifespan following bacteria elimination than that in strains with a lower level of commensal bacteria (Figure 3), suggesting that differential bacterial loads within the flies of each laboratory can give rise to differences in lifespan changes after bacterial elimination.

Under our experimental conditions, abundance of commensal microbes was a more important factor than the composition of the microbiota in lifespan determination of *Drosophila.* Ingestion of diluted old fly homogenate had an effect on lifespan that was similar to that of undiluted young fly homogenate, and the ingestion of young fly homogenate decreased the lifespan of flies in a dose-dependent manner (Figure 4). In addition, gnotobiotic flies colonized with a combination of four dominant bacteria showed that the abundance of commensal microbes exerted a significant influence on fly lifespan, whereas the composition of the microbiota exerted only a moderate effect on lifespan (Figure 5). Consistent with our results, several studies have reported on the effect of commensal microbial load on host lifespan. Flies fed homogenate from old flies have been shown to have a decreased lifespan compared to that in flies fed young fly homogenate [15]. Moreover, an increase in microbiota diversity has been correlated with an increase in bacterial load, resulting in a lifespan decrease [31]. In addition, mutant flies lacking the POU domain transcription factor Nub-PD had a shorter lifespan than control flies with a high diversity and high abundance microbiome [30], while flies treated with rapamycin exhibited delayed microbial expansion with age and had an extended lifespan [32].

Our study was the first to undertake a comparative analysis of the relative effects of microbiota load and microbiota composition on *Drosophila* lifespan. Our results show that microbiota load has a greater influence than microbiota composition on the lifespan of *Drosophila*; however, we cannot exclude the possibility that a specific bacterial species proliferates faster than others which could affect the lifespan of axenic flies. In addition, we cannot exclude the possibility that other factors can also affect the host’s lifespan, including microbial diversity and species specificity. There are several reports showing age-related increases in bacterial load and its deleterious effect on health, and there have been trials investigating the modulation of organismal health and lifespan by specific microbes. For example, *Bifidobacterium animalis lactis* has promoted longevity and reduced tumor incidence in mice [33], and *Lactobacillus salivarius* isolated from a centenarian's fecal samples extended the lifespan of *C. elegans* [34]. In addition, many bacteria have been shown to have species- or strain-specific effects on host health. *L. plantarum* was shown to affect systemic larval growth in *Drosophila* in a strain-specific manner [3]. Moreover, *L. plantarum* induces dNox dependent cellular ROS production, but that effect was not observed with other members of the microbiota [35]. In addition, *L. brevis* and *G. morbifer* can induce chronic DUOX activation via uracil production, while other commensal microbes cannot [36]. Consistent with those observations, our results showed that colonization of the most dominant microbes combinations at 10^8^ CFUs did not have a significant effect on lifespan, but inoculation of *Ap*+*Am* or *Lb+Lp+Ap* decreased lifespan (Figure 5C, Supplementary Figure 5, Supplementary Table 5–7, ANOVA, *p* < 0.0005; Tukey's HSD *vs.* Ax, Ax 85.59 ± 0.97 days; *Ap+Am* 79.08 ± 1.31 days, 7.61% decrease, *p* < 0.05; *Lb+Lp+Ap* 78.80 ± 1.28 days, 7.93% decrease, *p* < 0.05), even though the final concentrations of the inocula were equal (Figure 5D, Supplementary Table 10, Tukey's HSD *vs*. each group, *p* > 0.05). Moreover, although the bacteria decreased lifespan in all species combinations at high concentration (10^14^ CFUs), the extent of the decrease was different with the different microbe combinations (Figure 5C, Supplementary Table 5, Supplementary Tables 8, 9, ANOVA, *p* < 0.0005; Ax 76.68 ± 1.58 days; *Lb* 63.89 ± 1.42 days, 16.80% decrease, Tukey’s HSD test, *p* < 0.05; *Am* 65.17 ± 1.42 days, 15.01% decrease, Tukey’s HSD test, *p* < 0.005; *Lp+Ap* 66.72 ± 1.70 days, 12.99% decrease, Tukey’s HSD test, *p* < 0.005), indicating that there may be species-, strain-, or combination-specificity involved in the microbiota effects on lifespan regulation.

In addition, we cannot exclude the role of minor bacteria in lifespan regulation. In this study, we selected the four dominant species (the top two species in each age group) for our microbial combination study. Among the subdominant species, our 10-day-old flies contained *K. medellinensis*, and there was a high proportion presence of *L. pseudomesenteroides* in our 50-day-old flies. *K. medellinensis* (also called *Gluconacetobacter xylinus*) is reported to reduce triglyceride and glucose levels in *Drosophila* [37], and a reduced glucose level has been shown in flies with a low insulin-like peptide level, and such flies have been reported to live longer than control flies [38]. *Leuconostoc* negatively interacts with *Lactobacillus* indicating that the two genera occupy the same niche in *Drosophila* [39]; but whether *Leuconostoc* regulates lifespan along with *Lactobacillus* has not been reported. Taken collectively, the species-specific or compositional effects of the microbiome on the flies in this study may have been overshadowed by the stronger lifespan effect associated with bacterial abundance.

The mechanism of how commensal bacteria can regulate host lifespan is a fascinating study topic. There are some studies that have described this mechanism in relation to intestinal barrier dysfunction, ROS generation, intestinal stem cell dysplasia, and several lifespan-regulating pathways. Clark *et al.* showed that deleterious changes in a microbiota can induce intestinal barrier dysfunction and is a primary cause of mortality [15, 40]. In addition, the intracellular ROS level has been considered a critical cause of aging [41], and ROS generation induced by an increase in commensal or pathogenic microbes, as a defensive response, has been suggested to affect host lifespan [36, 42]. Furthermore, microbes are reported to regulate intestinal stem cell (ISC) proliferation by inducing ROS generation [23, 43, 44], and the elimination of the microbiota has induced the quiescent stage in ISCs and reduced the number of progenitor cells [43, 44]. Lastly, lifespan-regulating signaling pathways, including the insulin/IGF-1 signaling pathway and the target of rapamycin pathway, have been reported to be regulated by commensal microbes [3, 4].

Taken together, we suggest that when an axenic fly is generated for use in host-microbe interaction study, it should be noted that both egg bleaching and antibiotic treatment can have an adverse effect on the health of the *Drosophila*, but these adverse effects can be eliminated by allowing trans-generation of flies hatched from bleached eggs or by using a suitably diluted antibiotics. In this study, we demonstrated that the age-related increase in bacterial load more strongly affects the lifespan of *Drosophila* than that associated with changes in microbiota composition. Our results present a basic but deterministic connecting point in the relationship between commensal microbes and host lifespan.

## METHODS

### Fly husbandry and generation of axenic *D. melanogaster*

All experiments, except those related to the strain-specific longevity effect of commensal microbe elimination, were conducted using flies of the *D. melanogaster* wild-type strain *w^1118^* that were initially provided by the Bloomington Stock Center (Indiana University, USA) and have been adapting to our laboratory environment for 8 years. The enterobacteria *Wolbachia*, which can affect lifespan, was not present in the strains used in this study as was determined by PCR assay (data not shown). Flies were cultured and reared at 25ºC and 65% humidity on a 12:12 hour light:dark cycle. Sterile standard cornmeal-sugar-yeast (CSY) media (Supplementary Table 13) were used during culture and rearing of the flies. To produce the sterile CSY diet, the above-mentioned CSY medium was autoclaved at 120°C for 20 min, and all vials for food were exposed to UV light for 20 min on a clean bench. For the preparation of antibiotic-treated food, 640 μg/mL doxycycline (Sigma-Aldrich), 640 μg/mL ampicillin (Sigma-Aldrich), and 1 mg/mL kanamycin (Sigma-Aldrich) were added to sterile CSY media [12].

Axenic (Ax) flies were generated by bleaching the embryos. Embryos were collected for 12 h and were then dechorionated for 50 sec in 5% sodium hypochlorite solution (Wako, Japan), rinsed for 50 sec in 70% ethanol, and washed for 1 min in sterile distilled water [15]. Sterile embryos were transferred into sterile CSY food bottles on a clean bench. Eggs in an Ax condition were passed through repeated generations and became third-generation flies. All Ax flies were maintained on a clean bench and were transferred to fresh food every two days. The Ax conditions were confirmed by plating fly homogenate on plate count agar (PCA, Neogen Corporation, MI, USA) (Supplementary Table 13), and by 16S rRNA gene PCR using a bacterial 16S rRNA universal primer (27F and 1492R) provided by Macrogen (Seoul, South Korea).

### Bacteria culture

Conventionally, bacteria were cultured on PCA medium. *Lactobacillus* was grown on 5.5% MRS media (*Lactobacilli* MRS Broth, BD & Difco, MD, USA) and *Acetobacter* was grown on *Acetobacter*-selective media (see Supplementary Table 13). All microbes were incubated at 29°C.

### Quantitative analysis of bacteria

For CFU determination, 5 females were rinsed in 70% ethanol for 3 sec for surface decontamination and then homogenized in sterile distilled water. The homogenates were diluted as necessary and plated onto PCA media, MRS media, or *Acetobacter*-selective media. At least 5 replicates were established for each group. Data are presented as mean ± standard error of the mean (SEM) values. For 16S rRNA PCR, total genomic DNA from 45 surface-sterilized female flies was extracted by using a DNeasy Tissue Kit (Hilden, Germany) in accordance with the manufacturer’s instructions. The PCR assays were performed at a 60°C annealing temperature and for 40–60 cycles using taxon-specific 16S rRNA gene primers for *Lactobacillus* or *Acetobacter* designed using Primer3 software, as well as universal PCR primers. Sequences are presented in Supplementary Table 14.

### Identification of commensal microbes

For commensal microbe isolation, homogenates from 10- or 50-day-old female flies were plated on a PCA media plate. After incubation of a single colony at 29°C for 3 days, each colony was transferred to *Acetobacter*-selective or *Lactobacillus*-selective media broth. After culturing for 24 h, the cell walls of isolated microbes were broken down by bead beating using 0.1 mm diameter glass beads (BioSpec Products, Bartlesville, OK, USA). PCR assays were performed with a 55°C annealing temperature and 45 cycles with universal primers 27F and 1492R. PCR products were sequenced by using 16S sequencing (Macrogen Inc., South Korea) with universal primers 518F and 800R and then analyzed by using EzTaxon BLAST and NCBI BLAST. To determine the dominant commensal bacteria species in the gut of flies, 454 pyrosequencing analysis of the 16S rRNA gene was performed. The 16S rRNA gene amplicons from 100 dissected guts (comprising the Malpighian tubules but excluding the crop) from surface-sterilized females were analyzed by pyrosequencing using the 454 GS FLX Titanium Sequencing System (Roche, Brandford, CT, USA) at Chunlab Inc (South Korea). Phylogenetic relationships were determined by using EzTaxon BLAST and NCBI BLAST.

### Introduction of commensal microbes to axenic *D. melanogaster*

To feed fly homogenate to Ax fly, fly homogenates from surface sterile flies with 70% ethanol were seeded in CSY food vials. The Ax flies were transferred to new homogenate-containing vials three times for a week. To generate adult flies carrying a predetermined composition of bacteria (gnotobiotic flies), 100 μL of commensal bacterial cultures (10^3^, 10^8^, or 10^14^ CFUs) were added to sterilized food vials containing 2-day-old Ax flies. Every 2 days for 1 week, the flies were transferred to new sterile CSY food vials seeded with experiment-specific compositions of commensal bacteria. For commensal bacteria compositions of more than one species, each species component was added in equal parts to make up the total inoculum. The abundances of colonized microbes were identified *via* CFU testing 10 days after the initial infection.

### Lifespan assay

Newly eclosed adult female flies were collected for 2 days and were provided with a stabilizing time of 1 day with male flies. Mated female flies were randomly assigned to sterile CSY food vials to a final density of 20 flies per vial. Vials were changed every 2 days for new vials containing fresh sterile CSY food; at that time, dead flies were removed and the number was recorded. In the case of gnotobiotic flies, counting of dead flies was performed after bacterial seeding for 1 week. Ten replicate vials were established for each group (*n* = 200). Once a month, vials were spot checked for contamination by swabbing the food in the spent vials and plating on PCA-bearing culture plates.

### Statistical analysis

Log-rank tests were carried out to determine the statistical significance of the results of the survival analysis. The JMP statistical package (SAS, NC, USA) was used for the analyses. The statistical probabilities of the obtained CFU and OTU numbers were determined by using the two-sample *t*-test. ANOVA, Tukey’s HSD test, and Spearman’s correlation coefficients were derived by using R 3.5.1 software.

## Supplementary Material

Supplementary Figures

Supplementary Tables

Supplementary Table 3

Supplementary Table 12

## References

[1] Moran CP, Shanahan F. Gut microbiota and obesity: role in aetiology and potential therapeutic target. Best Pract Res Clin Gastroenterol. 2014; 28:585–97. 10.1016/j.bpg.2014.07.005 25194177

[2] Kostic AD, Xavier RJ, Gevers D. The microbiome in inflammatory bowel disease: current status and the future ahead. Gastroenterology. 2014; 146:1489–99. 10.1053/j.gastro.2014.02.009 24560869 PMC4034132

[3] Storelli G, Defaye A, Erkosar B, Hols P, Royet J, Leulier F. *Lactobacillus plantarum* promotes *Drosophila* systemic growth by modulating hormonal signals through TOR-dependent nutrient sensing. Cell Metab. 2011; 14:403–14. 10.1016/j.cmet.2011.07.012 21907145

[4] Shin SC, Kim SH, You H, Kim B, Kim AC, Lee KA, Yoon JH, Ryu JH, Lee WJ. *Drosophila* microbiome modulates host developmental and metabolic homeostasis via insulin signaling. Science. 2011; 334:670–74. 10.1126/science.1212782 22053049

[5] Diaz SA, Mooring EQ, Rens EG, Restif O. Association with pathogenic bacteria affects life-history traits and population growth in *Caenorhabditis elegans.* Ecol Evol. 2015; 5:1653–63. 10.1002/ece3.1461 25937908 PMC4409413

[6] Thaiss CA, Zeevi D, Levy M, Zilberman-Schapira G, Suez J, Tengeler AC, Abramson L, Katz MN, Korem T, Zmora N, Kuperman Y, Biton I, Gilad S, et al. Transkingdom control of microbiota diurnal oscillations promotes metabolic homeostasis. Cell. 2014; 159:514–29. 10.1016/j.cell.2014.09.048 25417104

[7] Yano JM, Yu K, Donaldson GP, Shastri GG, Ann P, Ma L, Nagler CR, Ismagilov RF, Mazmanian SK, Hsiao EY. Indigenous bacteria from the gut microbiota regulate host serotonin biosynthesis. Cell. 2015; 161:264–76. 10.1016/j.cell.2015.02.047 25860609 PMC4393509

[8] Houthoofd K, Braeckman BP, Lenaerts I, Brys K, De Vreese A, Van Eygen S, Vanfleteren JR. Axenic growth up-regulates mass-specific metabolic rate, stress resistance, and extends life span in *Caenorhabditis elegans.* Exp Gerontol. 2002; 37:1371–78. 10.1016/S0531-5565(02)00173-0 12559406

[9] Garsin DA, Villanueva JM, Begun J, Kim DH, Sifri CD, Calderwood SB, Ruvkun G, Ausubel FM. Long-lived *C. elegans* daf-2 mutants are resistant to bacterial pathogens. Science. 2003; 300:1921. 10.1126/science.1080147 12817143

[10] Han B, Sivaramakrishnan P, Lin CJ, Neve IAA, He J, Tay LWR, Sowa JN, Sizovs A, Du G, Wang J, Herman C, Wang MC. Microbial genetic composition tunes host longevity. Cell. 2017; 169:1249–1262.e13. 10.1016/j.cell.2017.05.036 28622510 PMC5635830

[11] Brummel T, Ching A, Seroude L, Simon AF, Benzer S. *Drosophila* lifespan enhancement by exogenous bacteria. Proc Natl Acad Sci USA. 2004; 101:12974–79. 10.1073/pnas.0405207101 15322271 PMC516503

[12] Ren C, Webster P, Finkel SE, Tower J. Increased internal and external bacterial load during *Drosophila* aging without life-span trade-off. Cell Metab. 2007; 6:144–52. 10.1016/j.cmet.2007.06.006 17681150

[13] Ridley EV, Wong AC, Westmiller S, Douglas AE. Impact of the resident microbiota on the nutritional phenotype of *Drosophila melanogaster.* PLoS One. 2012; 7:e36765. 10.1371/journal.pone.0036765 22586494 PMC3346728

[14] Petkau K, Parsons BD, Duggal A, Foley E. A deregulated intestinal cell cycle program disrupts tissue homeostasis without affecting longevity in *Drosophila.* J Biol Chem. 2014; 289:28719–29. 10.1074/jbc.M114.578708 25170078 PMC4192520

[15] Clark RI, Salazar A, Yamada R, Fitz-Gibbon S, Morselli M, Alcaraz J, Rana A, Rera M, Pellegrini M, Ja WW, Walker DW. Distinct shifts in microbiota composition during *Drosophila* aging impair intestinal function and drive mortality. Cell Rep. 2015; 12:1656–67. 10.1016/j.celrep.2015.08.004 26321641 PMC4565751

[16] Yamada R, Deshpande SA, Bruce KD, Mak EM, Ja WW. Microbes promote amino acid harvest to rescue undernutrition in *Drosophila.* Cell Rep. 2015; 10:865–72. 10.1016/j.celrep.2015.01.018 25683709 PMC4534362

[17] Galenza A, Hutchinson J, Campbell SD, Hazes B, Foley E. Glucose modulates *Drosophila* longevity and immunity independent of the microbiota. Biol Open. 2016; 5:165–73. 10.1242/bio.015016 26794610 PMC4823985

[18] Téfit MA, Leulier F. *Lactobacillus plantarum* favors the early emergence of fit and fertile adult *Drosophila* upon chronic undernutrition. J Exp Biol. 2017; 220:900–07. 10.1242/jeb.151522 28062579 PMC5358326

[19] Obata F, Fons CO, Gould AP. Early-life exposure to low-dose oxidants can increase longevity via microbiome remodelling in *Drosophila.* Nat Commun. 2018; 9:975. 10.1038/s41467-018-03070-w 29515102 PMC5841413

[20] Iatsenko I, Boquete JP, Lemaitre B. Microbiota-derived lactate activates production of reactive oxygen species by the intestinal NADPH oxidase nox and shortens Drosophila lifespan. Immunity. 2018; 49:929–942.e5. 10.1016/j.immuni.2018.09.017 30446385

[21] Han G, Lee HJ, Jeong SE, Jeon CO, Hyun S. Comparative analysis of *Drosophila melanogaster* gut microbiota with respect to host strain, sex, and age. Microb Ecol. 2017; 74:207–16. 10.1007/s00248-016-0925-3 28054304

[22] Wong CN, Ng P, Douglas AE. Low-diversity bacterial community in the gut of the fruitfly *Drosophila melanogaster.* Environ Microbiol. 2011; 13:1889–900. 10.1111/j.1462-2920.2011.02511.x 21631690 PMC3495270

[23] Guo L, Karpac J, Tran SL, Jasper H. PGRP-SC2 promotes gut immune homeostasis to limit commensal dysbiosis and extend lifespan. Cell. 2014; 156:109–22. 10.1016/j.cell.2013.12.018 24439372 PMC3928474

[24] Heintz C, Mair W. You are what you host: microbiome modulation of the aging process. Cell. 2014; 156:408–11. 10.1016/j.cell.2014.01.025 24485451 PMC3956044

[25] Smith P, Willemsen D, Popkes M, Metge F, Gandiwa E, Reichard M, Valenzano DR. Regulation of life span by the gut microbiota in the short-lived African turquoise killifish. eLife. 2017; 6:6. 10.7554/eLife.27014 28826469 PMC5566455

[26] Wostmann BS. Germ-free versus non-germ-free animals in gerontological research. 1968.

[27] Fast D, Duggal A, Foley E. The symbiont *Lactobacillus plantarum* causes intestinal pathogenesis in adult *Drosophila.* bioRxiv. 2016 10.1101/049981

[28] Li H, Qi Y, Jasper H. Preventing age-related decline of gut compartmentalization limits microbiota dysbiosis and extends lifespan. Cell Host Microbe. 2016; 19:240–53. 10.1016/j.chom.2016.01.008 26867182 PMC5106289

[29] Sannino DR, Dobson AJ, Edwards K, Angert ER, Buchon N. The *Drosophila melanogaster* gut microbiota provisions thiamine to its host. MBio. 2018; 9:e00155–18. 10.1128/mBio.00155-18 29511074 PMC5845000

[30] Dantoft W, Lundin D, Esfahani SS, Engström Y. The POU/Oct transcription factor Pdm1/nub is necessary for a beneficial gut microbiota and normal lifespan of *Drosophila.* J Innate Immun. 2016; 8:412–26. 10.1159/000446368 27231014 PMC6738862

[31] Gould A, Zhang V, Lamberti L, Jones E, Obadia B, Gavryushkin A, Carlson J, Beerenwinkel N, Ludington W. High-dimensional microbiome interactions shape host fitness. bioRxiv. 2017 10.1101/232959 PMC6304949 30510004

[32] Fan X, Liang Q, Lian T, Wu Q, Gaur U, Li D, Yang D, Mao X, Jin Z, Li Y, Yang M. Rapamycin preserves gut homeostasis during *Drosophila* aging. Oncotarget. 2015; 6:35274–83. 10.18632/oncotarget.5895 26431326 PMC4742104

[33] Matsumoto M, Kurihara S, Kibe R, Ashida H, Benno Y. Longevity in mice is promoted by probiotic-induced suppression of colonic senescence dependent on upregulation of gut bacterial polyamine production. PLoS One. 2011; 6:e23652. 10.1371/journal.pone.0023652 21858192 PMC3156754

[34] Zhao Y, Zhao L, Zheng X, Fu T, Guo H, Ren F. Lactobacillus salivarius strain FDB89 induced longevity in *Caenorhabditis elegans* by dietary restriction. J Microbiol. 2013; 51:183–88. 10.1007/s12275-013-2076-2 23625218

[35] Jones RM, Luo L, Ardita CS, Richardson AN, Kwon YM, Mercante JW, Alam A, Gates CL, Wu H, Swanson PA, Lambeth JD, Denning PW, Neish AS. Symbiotic *lactobacilli* stimulate gut epithelial proliferation via Nox-mediated generation of reactive oxygen species. EMBO J. 2013; 32:3017–28. 10.1038/emboj.2013.224 24141879 PMC3844951

[36] Lee KA, Kim SH, Kim EK, Ha EM, You H, Kim B, Kim MJ, Kwon Y, Ryu JH, Lee WJ. Bacterial-derived uracil as a modulator of mucosal immunity and gut-microbe homeostasis in Drosophila. Cell. 2013; 153:797–811. 10.1016/j.cell.2013.04.009 23663779

[37] Chaston JM, Newell PD, Douglas AE. Metagenome-wide association of microbial determinants of host phenotype in *Drosophila melanogaster.* MBio. 2014; 5:e01631–14. 10.1128/mBio.01631-14 25271286 PMC4196228

[38] Broughton SJ, Piper MD, Ikeya T, Bass TM, Jacobson J, Driege Y, Martinez P, Hafen E, Withers DJ, Leevers SJ, Partridge L. Longer lifespan, altered metabolism, and stress resistance in *Drosophila* from ablation of cells making insulin-like ligands. Proc Natl Acad Sci USA. 2005; 102:3105–10. 10.1073/pnas.0405775102 15708981 PMC549445

[39] Pais IS, Valente RS, Sporniak M, Teixeira L. Drosophila melanogaster establishes a species-specific mutualistic interaction with stable gut-colonizing bacteria. PLoS Biol. 2018; 16:e2005710. 10.1371/journal.pbio.2005710 29975680 PMC6049943

[40] Salazar AM, Resnik-Docampo M, Ulgherait M, Clark RI, Shirasu-Hiza M, Jones DL, Walker DW. Intestinal snakeskin limits microbial dysbiosis during aging and promotes longevity. iScience. 2018; 9:229–243. 10.1016/j.isci.2018.10.022 30419503 PMC6231084

[41] Barja G. The mitochondrial free radical theory of aging. Prog Mol Biol Transl Sci. 2014; 127:1–27. 10.1016/B978-0-12-394625-6.00001-5 25149212

[42] Jones RM, Desai C, Darby TM, Luo L, Wolfarth AA, Scharer CD, Ardita CS, Reedy AR, Keebaugh ES, Neish AS. *Lactobacilli* modulate epithelial cytoprotection through the Nrf2 pathway. Cell Rep. 2015; 12:1217–25. 10.1016/j.celrep.2015.07.042 26279578 PMC4640184

[43] Buchon N, Broderick NA, Chakrabarti S, Lemaitre B. Invasive and indigenous microbiota impact intestinal stem cell activity through multiple pathways in *Drosophila.* Genes Dev. 2009; 23:2333–44. 10.1101/gad.1827009 19797770 PMC2758745

[44] Broderick NA, Buchon N, Lemaitre B. Microbiota-induced changes in *drosophila melanogaster* host gene expression and gut morphology. MBio. 2014; 5:e01117–14. 10.1128/mBio.01117-14 24865556 PMC4045073

