# Supplementary material for: The role of commensal microbes in the lifespan of Drosophila melanogaster
Source: Aging (Albany NY). 2019 Jul 12;11(13):4611–40. doi: 10.18632/aging.102073 (PMC6660043; doi:10.18632/aging.102073)
Supplement: Supplementary Figures [file aging-11-102073-s004.pdf]

SUPPLEMENTARY MATERIALS

Supplementary Figures

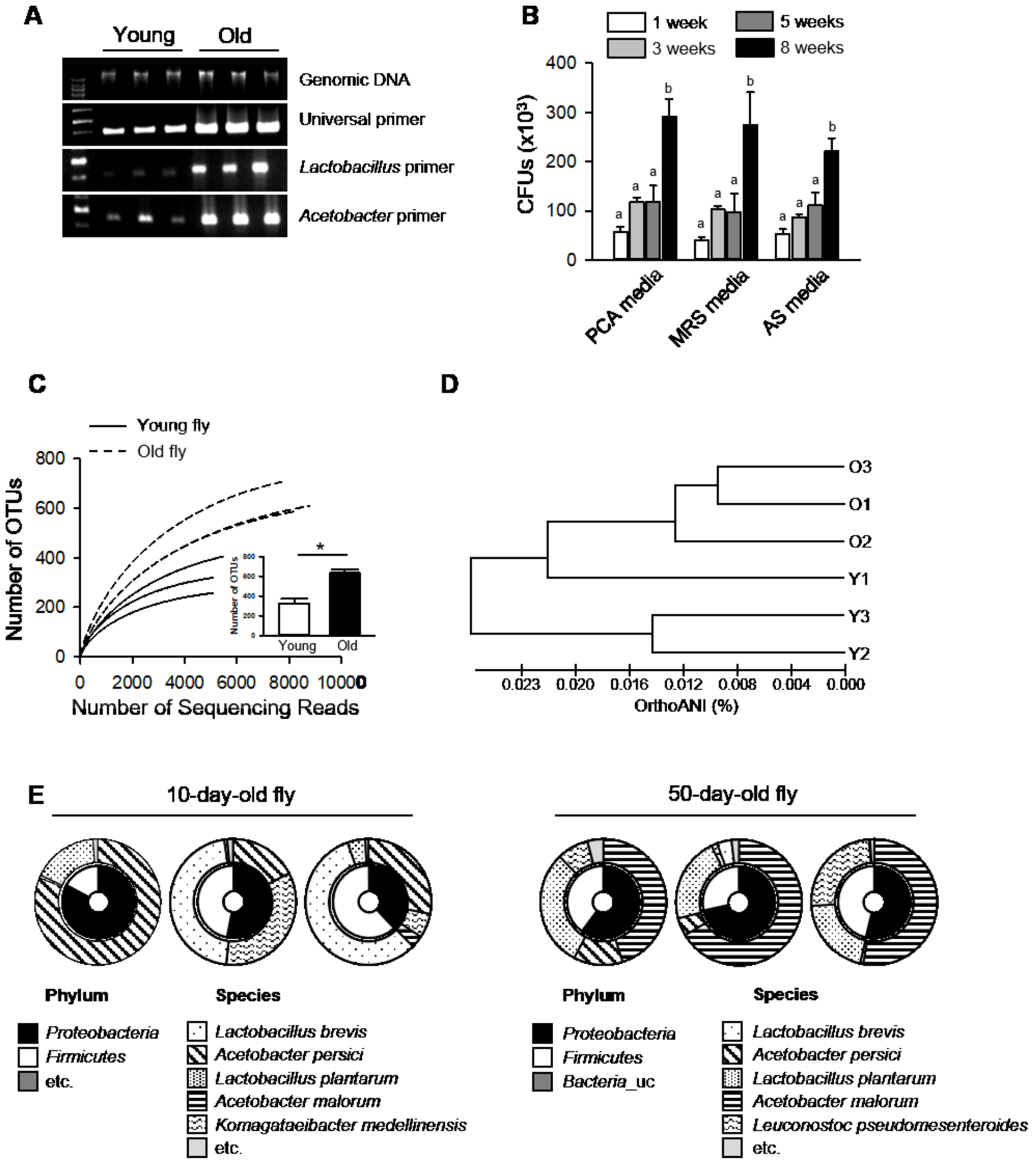

Supplementary Figure 1. Age-related changes in intestinal microbe flora. (**A**) PCR assay of microbial 16S rRNA amplified gene using universal (27F, 1492R), *Acetobacter*-, or *Lactobacillus*-specific primers. Microbial 16S rDNA gene sequences were amplified from genomic DNA extracted from 10-day-old flies (young; lanes 2 to 4) or 50-day-old flies (old; lanes 5 to 7). (**B**) The total number of CFU from 1-, 3-, 5-, and 8-week-old flies in PCA, MRS, or *Acetobacter*-selective media plates. Error bars represent the SEM. Different letters indicate significant differences between groups (ANOVA test, tukey's HSD test). (**C**) Rarefaction curves and the assigned number of operational taxonomic units (OTUs) from 454-pyrosequencing data. The inset shows the average number of OTUs in each group. Error bars represent the SEM. Asterisk indicates significant difference between the numbers of OTUs of young fly and old fly (*t*-test, **p* < 0.05). (**D**) Dendrogram using microbial flora analyzed by 454-pyrosequencing of 16S rRNA gene. (**E**) Double pie charts of three replicate about bacteria compositions of young or old fly guts. These charts show major phylum and species analyzed by 454-pyrosequencing of 16S rRNA gene.

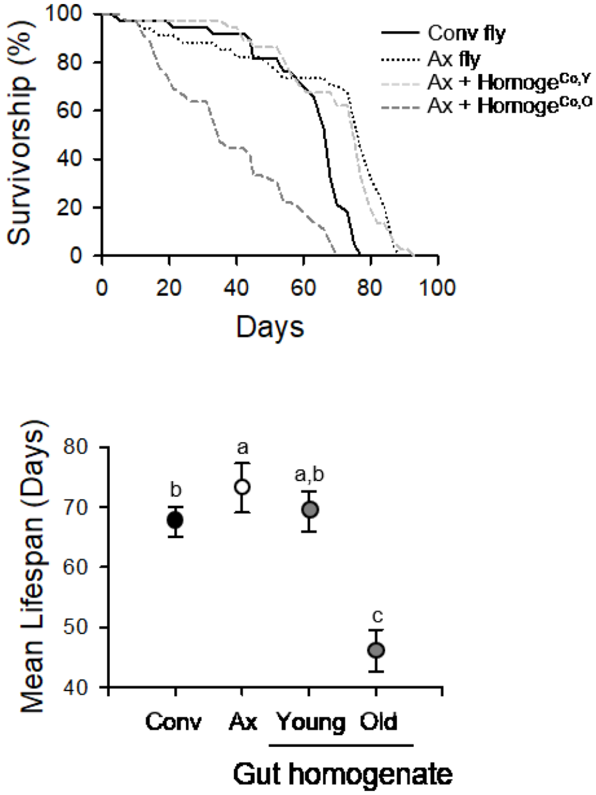

Supplementary Figure 2. Lifespan of flies fed gut homogenates from old conventional flies is shorter than that of flies fed gut homogenates from young conventional flies. The survival curve (upper) and mean lifespan (lower) of conventional (Conv) flies or axenic (Ax) flies fed gut homogenates from 10-day-old (young) or 50-day-old (old) flies. Solid line indicates Conv flies and dashed lines indicate Ax flies. Homoge^{Co,Y} indicates guts from young conventionally reared flies and Homoge^{Co,O} indicates guts from old conventionally reared flies. The lifespan of flies fed the gut from old flies was decreased more compared to that of flies fed the gut from young flies. Different letters indicate significant differences between groups (log-rank test, $p < 0.05$).

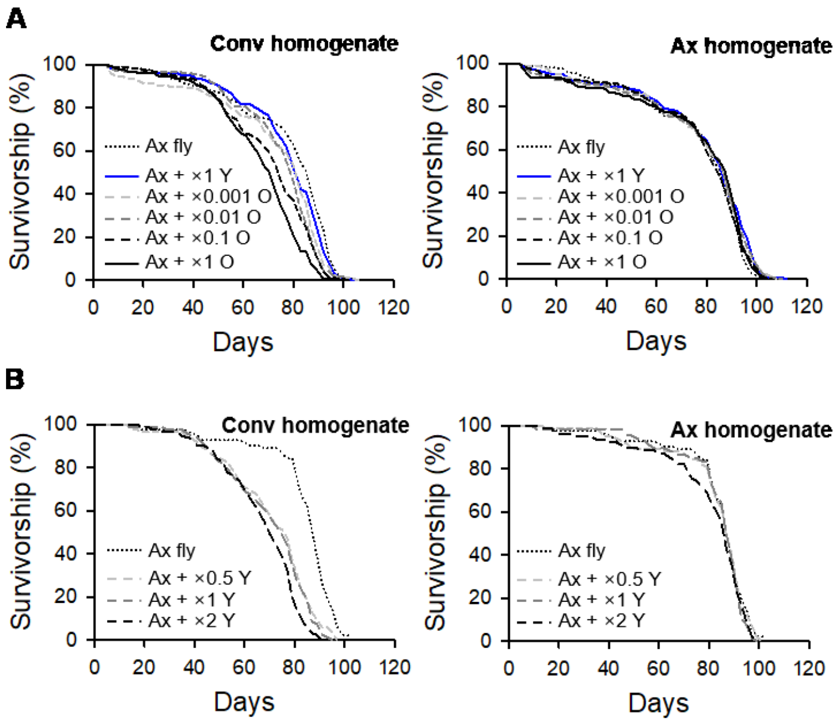

Supplementary Figure 3. Increased bacterial load shortens the lifespan of flies. (**A**) Survival curve of Ax flies after feeding diluted homogenate from old flies. (**B**) Survival curve of Ax flies after feeding concentrated homogenates from young flies.

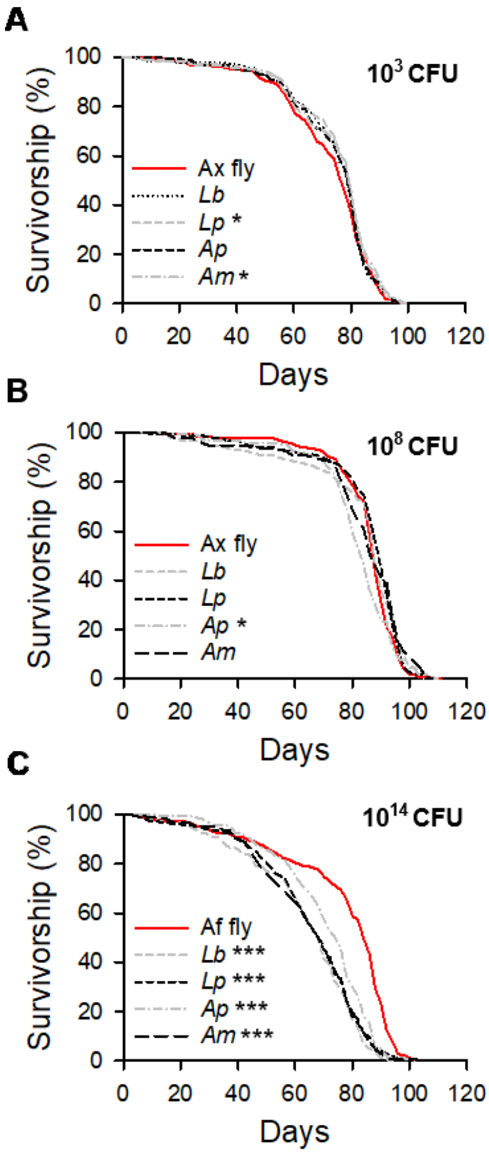

Supplementary Figure 4. Survival curve of flies inoculated with four dominant bacterial species at 10^3,10^8, or 10^{14} CFUs. The survival curve of flies inoculated with mono-association at 10^3 (**A**), 10^8 (**B**), or 10^{14} CFU (**C**). Asterisks indicate significant differences compared to Ax flies (log-rank test, *$p < 0.05$, **$p < 0.005$, ***$p < 0.0001$).

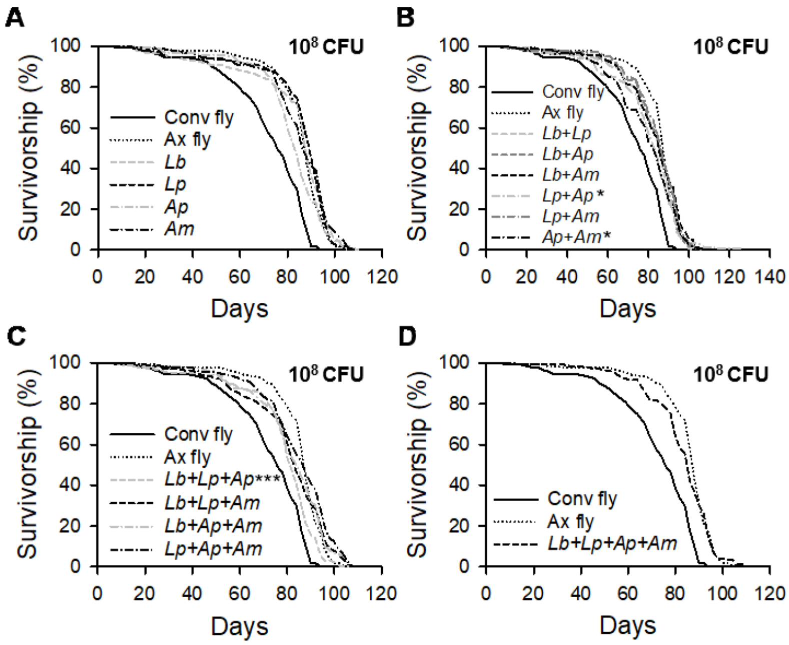

Supplementary Figure 5. Survival curve of flies inoculated with one of 2^4 combinations of the four dominant species at 10^8 CFUs. The survival curve of flies inoculated with mono- (**A**), dual- (**B**), triple- (**C**), and quadruple- (**D**) association at 10^8 CFU. Asterisks indicate significant differences compared to Ax flies (log-rank test, *$p < 0.05$, ***$p < 0.0001$).
