# Supplementary material for: The role of commensal microbes in the lifespan of Drosophila melanogaster
Source: Aging (Albany NY). 2019 Jul 12;11(13):4611–40. doi: 10.18632/aging.102073 (PMC6660043; doi:10.18632/aging.102073)
Supplement: Supplementary Tables [file aging-11-102073-s003.pdf]

Supplementary Tables

Supplementary Table 1. Lifespan of axenic flies.

Group		n	Mean Lifespan (Day)		Median Lifespan (Day)	Maximum Lifespan¶ (Day)	Log-rank		Wilcoxon	
Fly	Treat						χ^2	*p*-value	χ^2	*p*-value
Conv	DW†	191	59.92	± 1.52	70	76				
Ax	1G	179	53.83	± 1.31	58	66	38.85	<0.0001*	20.81	<0.0001*
Ax	2G	190	68.52	± 0.94	72	74	0.08	0.7710	8.91	0.00288*
Ax	3G	173	70.73	± 0.85	72	76	8.20	0.0042*	18.93	<0.0001*
Conv		180	65.41	± 1.54	72	80	20.42	<0.0001*	13.92	0.0002*
Ax†		191	73.00	± 1.40	76	84				
Ax + Feces^{Conv}		174	68.15	± 1.30	74	80	22.61	<0.0001*	13.72	0.0002*
Conv	×0 AB†	193	62.73	± 1.32	69	77				
Conv	×0.1 AB	184	68.93	± 1.16	71	81	12.33	0.0004*	11.02	0.0009*
Conv	×0.5 AB	186	67.84	± 0.99	69	77	2.86	0.0911	3.59	0.058
Conv	×1.0 AB	180	62.89	± 1.21	64	73	0.53	0.4659	0.48	0.4898
Ax	×0 AB†	183	77.17	± 1.32	83	87				
Ax	×0.1 AB	184	77.44	± 1.34	85	87	1.41	0.2347	0.71	0.4004
Ax	×0.5 AB	192	77.97	± 1.06	81	87	0.64	0.4238	0.24	0.6254
Ax	×1.0 AB	180	65.94	± 1.34	69	79	64.01	<0.0001*	63.64	<0.0001*
w^{1118}	Conv†	189	70.36	± 1.59	75	87	146.33	<0.0001*	146.66	<0.0001*
w^{1118}	Ax	188	92.88	± 0.94	96	100				
Oregon-R	Conv†	155	71.18	± 1.86	75	94	37.43	<0.0001*	26.41	<0.0001*
Oregon-R	Ax	182	83.15	± 1.69	90	98				
Canton-S	Conv†	188	79.26	± 1.30	82	92	6.60	0.0102*	2.22	0.136
Canton-S	Ax	183	81.66	± 1.47	85	96				

† These letters indicate a control for statistical analysis.
¶ Maximum lifespan means average of the last 25% of surviving flies.
* Asterisks indicate significant differences compared to control.

Supplementary Table 2. Richness and diversity estimation of the 16S rRNA gene libraries from the 454-pyrosequencing analysis.

Sample	Valid reads	OTUs[a]	Species richness indices		Species diversity indices		
			Ace	Chao1	JackKnife	Shannon	Simpson
Young #1	5,092	258	301	279	318	3	0
Young #2	5,473	406	523	484	543	3	0
Young #3	5,093	320	371	347	395	3	0
Old #1	7,704	707	844	781	890	4	0
Old #2	8,781	610	739	685	776	4	0
Old #3	8,294	589	697	656	743	4	0

[a] The operational taxonomic units (OTUs) were defined with pairwise 97% ID.

Please browse Full Text version to see the data of Supplementary Table 3:

Supplementary Table 3. Bacteria listed by 454-Pyrosequencing.

Supplementary Table 4. Lifespan of Ax flies fed Conv homogenate.

Group		n	Mean Lifespan (Day)		Median Lifespan (Day)	Maximum Lifespan[¶] (Day)	Log-rank		Wilcoxon	
Fly	Treat						χ^2	*p*-value	χ^2	*p*-value
Conv	DW	101	62.74	± 2.08	66	80	45.98	<0.0001*	51.93	<0.0001*
Ax	DW[†]	95	82.64	± 2.05	86	92				
	Homogenate[Co,Y]	90	75.93	± 2.09	80	88	4.61	<0.0318*	9.77	0.0018*
	Homogenate[Co,O]	103	64.46	± 2.32	70	84	43.12	<0.0001*	42.91	<0.0001*
	Homogenate[Ax,Y]	94	82.39	± 2.06	86	92	2.43	0.1192	0.40	0.5281
	Homogenate[Co,O]	99	79.14	± 2.21	82	90	0.49	0.4851	2.72	0.0991
Conv	DW	38	67.55	± 2.53	74	76	20.78	<0.0001*	11.26	0.0008*
Ax	DW[†]	34	73.18	± 4.11	83	90				
	Gut Homogenate[Co,Y]	38	75.47	± 2.89	82	85	0.02	0.8786	0.10	0.7470
	Gut Homogenate[Co,O]	36	44.22	± 3.27	41	60	38.41	<0.0001	26.56	<0.0001
Ax	DW	193	77.69	± 1.53	87	92	9.37	0.0022*	6.21	0.0127*
	×1 Young[†]	188	76.96	± 1.42	83	92				
	×0.001 Old	199	72.37	± 1.71	83	87	2.30	0.1293	2.57	0.1090
	×0.01 Old	199	74.33	± 1.32	80	87	8.35	0.0038*	5.47	0.0193*
	×0.1 Old	195	70.37	± 1.38	75	85	20.06	<0.0001*	17.46	<0.0001*
	×1 Old	189	66.74	± 1.34	73	80	55.63	<0.0001*	45.34	<0.0001*
Ax	DW	83	83.92	± 1.82	89	93	50.50	<0.0001*	45.52	<0.0001*
	×0.5 Young	86	70.52	± 1.99	77	83	0.61	0.4356	0.13	0.7164
	×1 Young[†]	84	69.98	± 1.87	75	83				
	×2 Young	95	67.28	± 1.63	70	79	5.40	0.0202*	2.23	0.1356

[†] These letters indicate a control for statistical analysis.

[¶] Maximum lifespan means average of the last 25% of surviving flies.

* Asterisks indicate significant differences compared to control.

Supplementary Table 5. Lifespan of Ax flies inoculated with dominant microbe.

Group			n	Mean Lifespan (Day)		Median Lifespan (Day)	Maximum Lifespan[¶] (Day)	Log-rank		Wilcoxon	
Conc	Fly	Inoculum						χ^2	*p*-value	χ^2	*p*-value
10^3 CFU	Ax	DW[†]	188	72.69	± 1.17	78	82				
		Lb	214	74.51	± 1.06	80	84	1.36	0.2428	1.95	0.1630
		Lp	193	75.40	± 1.14	80	84	4.99	0.0255*	4.66	0.0309*
		Ap	173	74.40	± 1.17	80	84	2.10	0.1475	1.69	0.1937
		Am	181	74.97	± 1.22	80	84	6.11	0.0134*	3.97	0.0463*
10^8 CFU	Ax	DW[†]	179	85.59	± 0.97	88	92				
	Mono	*Lb*	165	83.22	± 1.53	88	96	2.45	0.1173	0.42	0.5171
		Lp	154	85.89	± 1.27	90	94	3.50	0.0614	3.72	0.0539
		Ap	166	82.07	± 1.15	84	90	3.61	0.0575	11.59	0.0007*
		Am	173	84.14	± 1.38	88	96	2.89	0.0890	0.04	0.8468
	Dual	*Lb+Lp*	171	80.91	± 1.31	86	92	2.47	0.1162	5.73	0.0167*
		Lb+Ap	173	82.91	± 1.14	86	94	0.87	0.3515	2.57	0.1089
		Lb+Am	167	82.64	± 1.29	86	94	0.00	0.9579	2.40	0.1217
		Lp+Ap	173	80.54	± 1.30	84	90	5.72	0.0168*	15.50	<0.0001*
		Lp+Am	156	83.90	± 1.19	87	94	0.04	0.8477	0.97	0.3243
		Ap+Am	164	79.08	± 1.31	84	90	5.94	0.0148*	16.32	<0.0001*
	Triple	*Lb+Lp+Ap*	173	78.80	± 1.28	82	88	22.64	<0.0001	30.15	<0.0001*
		Lb+Lp+Am	168	80.72	± 1.41	84	94	0.25	0.6197	6.46	0.0111*
		Lb+Ap+Am	170	81.62	± 1.32	85	94	0.07	0.7988	4.72	0.0299*
		Lp+Ap+Am	156	84.49	± 1.32	88	96	3.65	0.0561	0.00	0.9577
	Quadruple	*Lb+Lp+Ap+Am*	169	83.00	± 1.11	86	94	0.95	0.329	4.78	0.0288
10^{14} CFU	Ax	DW[†]	180	76.68	± 1.58	84	90				
	Mono	*Lb*	185	63.89	± 1.42	68	78	92.74	<0.0001*	66.19	<0.0001*
		Lp	189	65.53	± 1.38	70	78	65.55	<0.0001*	54.68	<0.0001*
		Ap	176	70.56	± 1.26	76	84	39.09	<0.0001*	30.50	<0.0001*
		Am	173	65.17	± 1.42	70	78	65.64	<0.0001*	54.64	<0.0001*
	Dual	*Lb+Lp*	199	63.74	± 1.65	68	82	50.35	<0.0001*	46.33	<0.0001*
		Lb+Ap	190	68.25	± 1.56	76	84	39.02	<0.0001*	31.44	<0.0001*
		Lb+Am	187	68.11	± 1.62	76	84	33.92	<0.0001*	30.11	<0.0001*
		Lp+Ap	174	66.72	± 1.70	74	82	39.66	<0.0001*	36.25	<0.0001*
		Lp+Am	183	70.59	± 1.54	78	84	16.61	<0.0001*	19.05	<0.0001*
		Ap+Am	183	71.91	± 1.51	78	86	13.60	0.0002*	13.00	0.0003*
	Triple	*Lb+Lp+Ap*	192	67.64	± 1.51	72	82	52.62	<0.0001*	40.14	<0.0001*
		Lb+Lp+Am	189	67.72	± 1.50	74	84	41.68	<0.0001*	35.58	<0.0001*
		Lb+Ap+Am	183	65.84	± 1.45	70	80	62.75	<0.0001*	49.17	<0.0001*
		Lp+Ap+Am	181	68.67	± 1.56	74	84	17.92	<0.0001*	22.87	<0.0001*
	Quadruple	*Lb+Lp+Ap+Am*	190	69.34	± 1.67	78	86	20.53	<0.0001*	17.66	<0.0001*

[†] These letters indicate a control for statistical analysis.

[¶] Maximum lifespan means average of the last 25% of surviving flies.

* Asterisks indicate significant differences compared to control.

Supplementary Table 6. Log-rank test result of survival with a combination of microbes (10^8 CFUs).

p-value (10^8 CFUs)		Conv	Ax	Mono-association				Dual-association						Triple-association				Quadruple-association
				Lb	***Lp***	***Ap***	***Am***	***Lb+Lp***	***Lb+Ap***	***Lb+Am***	***Lp+Ap***	***Lp+Am***	***Ap+Am***	***Lb+Lp +Ap***	***Lb+Lp +Am***	***Lb+Ap +Am***	***Lp+Ap +Am***	***Lb+Lp +Ap+Am***
Conv		1	<0.0001	<0.0001	<0.0001	<0.0001	<0.0001	<0.0001	<0.0001	<0.0001	<0.0001	<0.0001	<0.0001	<0.0001	<0.0001	<0.0001	<0.0001	<0.0001
Ax		<0.0001	1	0.1173	0.0614	0.0575	0.089	0.1162	0.3515	0.9579	0.0168	0.8477	0.0148	<0.0001	0.6197	0.7988	0.0561	0.329
Mono-association	*Lb*	<0.0001	0.1173	1	0.9847	0.0027	0.9243	0.0036	0.0138	0.1617	0.0019	0.0849	0.0006	<0.0001	0.0949	0.1308	0.573	0.0310
	Lp	<0.0001	0.0614	0.9847	1	0.0014	0.8059	0.0021	0.0071	0.1658	0.0002	0.0688	0.0004	<0.0001	0.0961	0.0969	0.4865	0.0163
	Ap	<0.0001	0.0575	0.0027	0.0014	1	0.0013	0.9144	0.4652	0.1869	0.8305	0.202	0.4248	0.0216	0.2611	0.1923	0.0022	0.3033
	Am	<0.0001	0.089	0.9243	0.8059	0.0013	1	0.0008	0.0044	0.0612	0.0017	0.052	0.0002	<0.0001	0.0948	0.1009	0.6675	0.0394
Dual-association	*Lb+Lp*	<0.0001	0.1162	0.0036	0.0021	0.9144	0.0008	1	0.577	0.0951	0.4639	0.1574	0.4751	0.0119	0.234	0.2266	0.0008	0.5315
	Lb+Ap	<0.0001	0.3515	0.0138	0.0071	0.4652	0.0044	0.577	1	0.1803	0.2086	0.4883	0.2246	0.0009	0.5509	0.5006	0.0047	0.8503
	Lb+Am	<0.0001	0.9579	0.1617	0.1658	0.1869	0.0612	0.0951	0.1803	1	0.0765	0.7014	0.0323	0.0001	0.9461	0.9233	0.0453	0.5918
	Lp+Ap	<0.0001	0.0168	0.0019	0.0002	0.8305	0.0017	0.4639	0.2086	0.0765	1	0.0943	0.6897	0.0683	0.2806	0.1923	0.0027	0.2054
	Lp+Am	<0.0001	0.8477	0.0849	0.0688	0.202	0.052	0.1574	0.4883	0.7014	0.0943	1	0.0571	0.0001	0.907	0.8301	0.0234	0.6719
	Ap+Am	<0.0001	0.0148	0.0006	0.0004	0.4248	0.0002	0.4751	0.2246	0.0323	0.6897	0.0571	1	0.1428	0.0908	0.0757	0.0002	0.1311
Triple-association	*Lb+Lp+Ap*	<0.0001	<0.0001	<0.0001	<0.0001	0.0216	<0.0001	0.0119	0.0009	0.0001	0.0683	0.0001	0.1428	1	0.0012	0.0007	<.0001	0.0009
	Lb+Lp +Am	<0.0001	0.6197	0.0949	0.0961	0.2611	0.0948	0.234	0.5509	0.9461	0.2806	0.907	0.0908	0.0012	1	0.9247	0.0548	0.9211
	Lb+Ap +Am	<0.0001	0.7988	0.1308	0.0969	0.1923	0.1009	0.2266	0.5006	0.9233	0.1923	0.8301	0.0757	0.0007	0.9247	1	0.0592	0.7937
	Lp+Ap +Am	<0.0001	0.0561	0.573	0.4865	0.0022	0.6675	0.0008	0.0047	0.0453	0.0027	0.0234	0.0002	<.0001	0.0548	0.0592	1	0.0338
Quadruple-association	*Lb+Lp +Ap +Am*	<0.0001	0.329	0.0310	0.0163	0.3033	0.0394	0.5315	0.8503	0.5918	0.2054	0.6719	0.1311	0.0009	0.9211	0.7937	0.0338	1

Supplementary Table 7. ANOVA and Tukey's HSD test results for fly lifespans treated with a combination of microbes (10^8 CFUs).

**ANOVA**					
	**Sum of sqrs**	**Df**	**Mean Square**	**F**	***p*-value**
**Between groups**	11103	15	740.2	2.734	0.000343
**Residuals**	720387	2661	270.7		

Tukey's HSD (Honestly Significant Difference) test

***p*-value (10^8 CFUs)**		**Ax**	**Mono-association**				**Dual-association**						**Triple-association**				**Quadruple-association**
			Lb	***Lp***	***Ap***	***Am***	***Lb+Lp***	***Lb+Ap***	***Lb+Am***	***Lp+Ap***	***Lp+Am***	***Ap+Am***	***Lb+Lp +Ap***	***Lb+Lp +Am***	***Lb+Ap +Am***	***Lp+Ap +Am***	***Lb+Lp +Ap+Am***
Ax		1	0.994681	1.000000	0.833558	0.999983	0.357371	0.979056	0.955049	0.226635	0.999914	0.022988	0.010642	0.295254	0.659396	1.000000	0.985834
Mono-association	*Lb*	0.994681	1	0.987869	1	1	0.996339	1	1	0.982957	1	0.635314	0.492619	0.991913	0.999955	0.999999	1
	Lp	1	0.9878685	1	0.782016	0.999883	0.316062	0.961808	0.927661	0.199458	0.999595	0.020921	0.009965	0.260160	0.600623	0.999996	0.972496
	Ap	0.833558	0.9999995	0.782016	1	0.998904	0.999999	1	1	0.999973	0.999811	0.958406	0.904932	0.999995	1	0.995235	1
	Am	0.999983	1	0.999883	0.998904	1	0.909980	0.999998	0.999979	0.807035	1	0.257200	0.161460	0.867783	0.990172	1	1
Dual-association	*Lb+Lp*	0.357371	0.9963387	0.316062	0.999999	0.909980	1	0.999204	0.999875	1	0.960528	0.999755	0.998445	1	1	0.843913	0.998743
	Lb+Ap	0.979056	1	0.961808	1	0.999998	0.999204	1	1	0.99456	1	0.742384	0.606355	0.997838	0.999997	0.999965	1
	Lb+Am	0.955049	1	0.927661	1	0.999979	0.999875	1	1	0.99868	0.999999	0.842333	0.730064	0.999568	1	0.999778	1
	Lp+Ap	0.226635	0.9829566	0.199458	0.999973	0.807035	1	0.99456	0.998677	1	0.897808	0.999986	0.999836	1	1	0.715293	0.992377
	Lp+Am	0.999914	1	0.999595	0.999811	1	0.960528	1	0.999999	0.897808	1	0.383468	0.263260	0.936032	0.997351	1	1
	Ap+Am	0.022988	0.6353135	0.020921	0.958406	0.2572	0.999755	0.742384	0.842333	0.999986	0.383468	1	1	0.999942	0.990361	0.195019	0.715643
Triple-association	*Lb+Lp+Ap*	0.010642	0.4926191	0.009965	0.904932	0.16146	0.998445	0.606355	0.730064	0.999836	0.26326	1	1	0.999514	0.970238	0.119008	0.577168
	Lb+Lp +Am	0.295254	0.9919125	0.26016	0.999995	0.867783	1	0.997838	0.999568	1	0.936032	0.999942	0.999514	1	1	0.788867	0.996799
	Lb+Ap +Am	0.659396	0.9999554	0.600623	1	0.990172	1	0.999997	1	1	0.997351	0.990361	0.970238	1	1	0.972992	0.999993
	Lp+Ap +Am	1	0.9999985	0.999996	0.995235	1	0.843913	0.999965	0.999778	0.715293	1	0.195019	0.119008	0.788867	0.972992	1	0.999985
Quadruple-association	*Lb+Lp +Ap +Am*	0.985834	1	0.972496	1	1	0.998743	1	1	0.992377	1	0.715643	0.577168	0.996799	0.999993	0.999985	1

Supplementary Table 8. Log-rank test result of survival with a combination of microbes (10^{14} CFUs).

p-value (10^{14} CFUs)		Conv	Ax	Mono-association				Dual-association						Triple-association				Quadruple-association
				Lb	***Lp***	***Ap***	***Am***	***Lb+Lp***	***Lb+Ap***	***Lb+Am***	***Lp+Ap***	***Lp+Am***	***Ap+Am***	***Lb+Lp +Ap***	***Lb+Lp +Am***	***Lb+Ap +Am***	***Lp+Ap +Am***	***Lb+Lp +Ap+Am***
Conv		1	<0.0001	<0.0001	0.0062	0.8377	0.0058	0.1285	0.771	0.9889	0.5488	0.1516	0.0397	0.2226	0.5122	0.0247	0.408	0.1009
Ax		<0.0001	1	<0.0001	<0.0001	<0.0001	<0.0001	<0.0001	<0.0001	<0.0001	<0.0001	<0.0001	0.0002	<0.0001	<0.0001	<0.0001	<0.0001	<0.0001
Mono-association	*Lb*	<0.0001	<0.0001	1	0.2541	0.0002	0.3553	0.0330	<0.0001	<0.0001	0.0015	<0.0001	<0.0001	0.0043	0.0009	0.0780	<0.0001	<0.0001
	Lp	0.0062	<0.0001	0.2541	1	0.0098	0.8710	0.4144	0.0162	0.0067	0.0560	<0.0001	<0.0001	0.0959	0.0379	0.6300	0.0018	0.0002
	Ap	0.8377	<0.0001	0.0002	0.0098	1	0.0133	0.1600	0.9552	0.7885	0.6910	0.1304	0.0176	0.3251	0.6974	0.0486	0.3251	0.0494
	Am	0.0058	<0.0001	0.3553	0.8710	0.0133	1	0.2910	0.0081	0.0047	0.0451	<.0001	<0.0001	0.0949	0.0318	0.5329	0.0018	<.0001
Dual-association	*Lb+Lp*	0.1285	<0.0001	0.0330	0.4144	0.1600	0.2910	1	0.1752	0.0847	0.2913	0.0023	0.0002	0.5869	0.3166	0.5590	0.0153	0.0020
	Lb+Ap	0.771	<0.0001	<0.0001	0.0162	0.9552	0.0081	0.1752	1	0.7121	0.6413	0.0766	0.0171	0.2926	0.7354	0.0491	0.2346	0.1067
	Lb+Am	0.9889	<0.0001	<0.0001	0.0067	0.7885	0.0047	0.0847	0.7121	1	0.4702	0.1447	0.0546	0.1859	0.5345	0.0223	0.4177	0.2066
	Lp+Ap	0.5488	<0.0001	0.0015	0.0560	0.6910	0.0451	0.2913	0.6413	0.4702	1	0.0526	0.0091	0.5111	0.9218	0.1221	0.2115	0.0359
	Lp+Am	0.1516	<0.0001	<0.0001	<0.0001	0.1304	<0.0001	0.0023	0.0766	0.1447	0.0526	1	0.7537	0.0078	0.0421	0.0003	0.4952	0.8921
	Ap+Am	0.0397	0.0002	<0.0001	<0.0001	0.0176	<0.0001	0.0002	0.0171	0.0546	0.0091	0.7537	1	0.0006	0.0087	<.0001	0.3796	0.3732
Triple-association	*Lb+Lp+Ap*	0.2226	<0.0001	0.0043	0.0959	0.3251	0.0949	0.5869	0.2926	0.1859	0.5111	0.0078	0.0006	1	0.5501	0.2559	0.0688	0.0031
	Lb+Lp +Am	0.5122	<0.0001	0.0009	0.0379	0.6974	0.0318	0.3166	0.7354	0.5345	0.9218	0.0421	0.0087	0.5501	1	0.1200	0.1612	0.0472
	Lb+Ap +Am	0.0247	<0.0001	0.0780	0.6300	0.0486	0.5329	0.5590	0.0491	0.0223	0.1221	0.0003	<0.0001	0.2559	0.1200	1	0.0042	0.0002
	Lp+Ap +Am	0.408	<0.0001	<0.0001	0.0018	0.3251	0.0018	0.0153	0.2346	0.4177	0.2115	0.4952	0.3796	0.0688	0.1612	0.0042	1	0.7844
Quadruple-association	*Lb+Lp +Ap +Am*	0.1009	<0.0001	<0.0001	0.0002	0.0494	<0.0001	0.0020	0.1067	0.2066	0.0359	0.8921	0.3732	0.0031	0.0472	0.0002	0.7844	1

Supplementary Table 9. ANOVA and Tukey's HSD test result of lifespan with a combination of microbes (10^{14} CFUs).

ANOVA

	**Sum of sqrs**	**Df**	**Mean Square**	**F**	***p*-value**
**Between groups**	31614	15	2107.6	2.962	0.000102
**Residuals**	2254285	3168	711.6		

Tukey's HSD (Honestly Significant Difference) test

***p*-value (10^{14} CFUs)**		**Ax**	**Mono-association**				**Dual-association**						**Triple-association**				**Quadruple-association**
			Lb	***Lp***	***Ap***	***Am***	***Lb+Lp***	***Lb+Ap***	***Lb+Am***	***Lp+Ap***	***Lp+Am***	***Ap+Am***	***Lb+Lp +Ap***	***Lb+Lp +Am***	***Lb+Ap +Am***	***Lp+Ap +Am***	***Lb+Lp +Ap+Am***
Ax		1	0.018132	0.355422	0.398274	0.000243	0.764823	0.973670	0.823822	0.003995	0.956063	0.998188	0.982943	0.882279	0.074832	0.486508	0.998588
Mono-association	*Lb*	0.018132	1	0.999582	0.999181	0.999752	0.963559	0.725966	0.940439	1	0.787346	0.456969	0.675366	0.901177	1	0.997407	0.437559
	Lp	0.355422	0.999582	1	1	0.774434	1	0.999428	1	0.986822	0.999808	0.987151	0.998802	1	1	1	0.984703
	Ap	0.398274	0.999181	1	1	0.733675	1	0.999717	1	0.980349	0.999914	0.991688	0.999367	1	0.999998	1	0.989958
	Am	0.000243	0.999752	0.774434	0.733675	1	0.365608	0.105301	0.301995	1	0.135908	0.034791	0.085921	0.235038	0.987473	0.647719	0.031852
Dual-association	*Lb+Lp*	0.764823	0.963559	1	1	0.365608	1	1	1	0.814224	1	1	1	1	0.998370	1	0.999933
	Lb+Ap	0.97367	0.725966	0.999428	0.999717	0.105301	1	1	1	0.433435	1	1	1	1	0.939380	0.999940	1
	Lb+Am	0.823822	0.940439	0.999999	1	0.301995	1	1	1	0.753798	1	1	1	1	0.996122	1	0.999984
	Lp+Ap	0.003995	1	0.986822	0.980349	1	0.814224	0.433435	0.753798	1	0.503566	0.209144	0.382443	0.672628	0.999984	0.960918	0.196513
	Lp+Am	0.956063	0.787346	0.999808	0.999914	0.135908	1	1	1	0.503566	1	1	1	1	0.962067	0.999985	1
	Ap+Am	0.998188	0.456969	0.987151	0.991688	0.034791	0.999954	1	0.99999	0.209144	1	1	1	1	0.772048	0.996793	1
Triple-association	*Lb+Lp+Ap*	0.982943	0.675366	0.998802	0.999367	0.085921	1	1	1	0.382443	1	1	1	1	0.916661	0.999844	1
	Lb+Lp +Am	0.882279	0.901177	0.999993	0.999998	0.235038	1	1	1	0.672628	1	0.999999	1	1	0.990479	1	1
	Lb+Ap +Am	0.074832	1	1	0.999998	0.987473	0.99837	0.93938	0.996122	0.999984	0.962067	0.772048	0.916661	0.990479	1	0.999985	0.755034
	Lp+Ap +Am	0.486508	0.997407	1	1	0.647719	1	0.99994	1	0.960918	0.999985	0.996793	0.999844	1	0.999985	1	0.995999
Quadruple-association	*Lb+Lp +Ap +Am*	0.998588	0.437559	0.984703	0.989958	0.031852	0.999933	1	0.999984	0.196513	1	1	1	0.999998	0.755034	0.996	1

Supplementary Table 10. ANOVA and Tukey’s HSD test result of CFUs from fly's body with a combination of microbes (10^8 CFUs).

ANOVA

	Sum of sqrs	Df	Mean Square	F	*p*-value
**Between groups**	749375	14	53527	2.838	0.00257
**Residuals**	1131742	60	18862		

Tukey’s HSD (Honestly Significant Difference) test

p-value (10^8 CFUs)		Mono-association				Dual-association						Triple-association				Quadruple-association
		Lb	*Lp*	*Ap*	*Am*	*Lb+Lp*	*Lb+Ap*	*Lb+Am*	*Lp+Ap*	*Lp+Am*	*Ap+Am*	*Lb+Lp +Ap*	*Lb+Lp +Am*	*Lb+Ap +Am*	*Lp+Ap +Am*	*Lb+Lp +Ap+Am*
Mono-association	*Lb*	1	1	0.1774	0.9998	1	0.0051	0.9997	0.9999	0.9641	0.9981	0.3202	0.9999	0.9547	0.6564	0.8382
	Lp	1	1	0.2586	1	1	0.0091	0.9999	1	0.9880	0.9997	0.4342	1	0.9838	0.7749	0.9160
	Ap	0.1774	0.2586	1	0.7178	0.2978	0.9926	0.7470	0.5230	0.9754	0.8495	1	0.6875	0.9813	1	0.9984
	Am	0.9998	1	0.7178	1	1	0.0701	1	1	1	1	0.8809	1	1	0.9913	0.9994
Dual-association	*Lb+Lp*	1	1	0.2978	1	1	0.0114	0.9999	1	0.9929	0.9999	0.4845	1	0.9901	0.8172	0.9390
	Lb+Ap	0.0051	0.0091	0.9926	0.0701	0.0114	1	0.0788	0.0326	0.2946	0.1235	0.9547	0.0622	0.3202	0.7325	0.5283
	Lb+Am	0.9997	0.9999	0.7470	1	0.9999	0.0788	1	1	1	1	0..8994	1	0.9999	0.9938	0.9996
	Lp+Ap	0.9999	1	0.5230	1	1	0.0326	1	1	0.9998	0.9999	0.7276	1	0.9996	0.9529	0.9922
	Lp+Am	0.9641	0.9880	0.9754	1	0.9929	0.2946	1	0.9998	1	1	0.9971	1	1	1	1
	Ap+Am	0.9981	0.9997	0.8495	1	0.9999	0.1235	1	0.9999	1	1	0.9547	1	1	0.9987	1
Triple-association	*Lb+Lp+Ap*	0.3202	0.4342	1	0.8809	0.4845	0.9547	0.8994	0.7276	0.9971	0.9547	1	0.8605	0.9981	1	1
	Lb+Lp +Am	0.9999	1	0.6875	1	1	0.0622	1	1	1	1	0.8605	1	1	0.9880	0.9990
	Lb+Ap +Am	0.9547	0.9838	0.9813	1	0.9901	0.3202	0.9999	0.9996	1	1	0.9981	1	1	1	1
	Lp+Ap +Am	0.6564	0.7749	1	0.9913	0.8172	0.7325	0.9938	0.9529	1	0.9987	1	0.9880	1	1	1
Quadruple-association	*Lb+Lp +Ap +Am*	0.8382	0.9160	0.9984	0.9994	0.9390	0.5283	0.9996	0.9922	1	1	1	0.9990	1	1	1

Supplementary Table 11. ANOVA and Tukey's HSD test result of CFUs from fly's body with a combination of microbes (10^{14} CFUs).

ANOVA					
	**Sum of sqrs**	**Df**	**Mean Square**	**F**	**p-value**
**Between groups**	668496	14	47750	4.901	< 0.0001
**Residuals**	584590	60	9743		

Tukey's HSD (Honestly Significant Difference) test

p-value (10^{14} CFUs)		**Mono-association**				**Dual-association**						**Triple-association**			**Quadruple-association**
		Lb	*Lp*	*Ap*	*Am*	*Lb+Lp*	*Lb+Ap*	*Lb+Am*	*Lp+Ap*	*Lp+Am*	*Ap+Am*	*Lb+Lp +Ap*	*Lb+Lp +Am*	*Lb+Ap +Am*	*Lp+Ap +Am*	*Lb+Lp +Ap+Am*
Mono-association	*Lb*	1	1	0.1824	0.9255	0.9832	0.3352	0.9705	0.2053	0.8912	0.9999	0.9958	<0.0001	0.1450	0.1672	0.4407
	Lp	1	1	0.2531	0.9650	0.9945	0.4354	0.9890	0.2817	0.9438	1	0.9990	<0.0001	0.2053	0.2339	0.5506
	Ap	0.1824	0.2531	1	0.9917	0.9535	1	0.9718	1	0.9960	0.7046	0.8943	0.0652	1	1	1
	Am	0.9255	0.9650	0.9917	1	1	0.9995	1	0.9945	1	0.9999	1	0.0012	0.9832	0.9890	0.9999
Dual-association	*Lb+Lp*	0.9832	0.9945	0.9535	1	1	0.9930	1	0.9650	1	1	1	0.0004	0.9255	0.9438	0.9983
	Lb+Ap	0.3352	0.4354	1	0.9995	0.993	1	0.9968	1	0.9998	0.8779	0.9754	0.0278	1	1	1
	Lb+Am	0.9705	0.9890	0.9718	1	1	0.9968	1	0.9796	1	1	1	0.0006	0.9517	0.9650	0.9994
	Lp+Ap	0.2053	0.2817	1	0.9945	0.9650	1	0.9796	1	0.9976	0.7406	0.9150	0.0563	1	1	1
	Lp+Am	0.8912	0.9438	0.9960	1	1	0.9998	1	0.9976	1	0.9998	1	0.0016	0.9912	0.9945	1
	Ap+Am	0.9999	1	0.7046	0.9999	1	0.8779	1	0.7406	0.9998	1	1	0.0006	0.6344	0.6781	0.9374
Triple-association	*Lb+Lp+Ap*	0.9958	0.9990	0.8943	1	1	0.9754	1	0.9150	1	1	1	0.0002	0.8485	0.8779	0.9917
	Lb+Lp +Am	< 0.0001	<0.0001	0.0652	0.0012	0.0004	0.0278	0.0006	0.0563	0.0016	0.0006	0.0002	1	0.0850	0.0723	0.0170
	Lb+Ap +Am	0.1450	0.2053	1	0.9832	0.9255	1	0.9517	1	0.9912	0.6344	0.8485	0.0850	1	1	1
	Lp+Ap +Am	0.1672	0.2339	1	0.9890	0.9438	1	0.965	1	0.9945	0.6781	0.8779	0.0723	1	1	1
Quadruple-association	*Lb+Lp +Ap +Am*	0.4407	0.5506	1	0.9999	0.9983	1	0.9994	1	1	0.9374	0.9917	0.0170	1	1	1

Please browse Full Text version to see the data of Supplementary Table 12:

Supplementary Table 12. Summary of axenic fly lifespan data in previous reports.

Supplementary Table 13. Composition of fly husbandry food and bacteria incubation media.

Food	Composition
Cornmeal-sugar-yeast (CSY) media	5.2% cornmeal<br>11% sugar<br>2.5% instant yeast<br>0.5% propionic acid<br>0.04% methyl-4-hydroxybenzoate<br>1% agar
Plate count agar (PCA)	0.5% tryptone<br>0.25% yeast extract<br>0.1% glucose<br>1.5% bacto agar
Lactobacillus-selective (MRS) media	1% peptone<br>1% beef extract<br>0.5% yeast extract<br>2% dextrose<br>0.1% polysorbate<br>0.2% ammonium citrate<br>0.5% sodium acetate<br>0.01% magnesium sulfate<br>0.005% manganese sulfate<br>0.2% dipotassium phosphate<br>1.5% bacto agar (BD & Difco)
Acetobacter-selective media	2.5% D-mannitol (BD & Difco)<br>0.5% yeast extract (BD & Difco)<br>0.3% peptone (BD & Difco)<br>1.5% bacto agar

Supplementary Table 14. 16S rRNA PCR primer sequences.

	Forward (5'-3')	Reverse (5'-3')
Lactobacillus-selective primer	GCAAGGCTGAAACTCAAAGG	TTCATGTAGGCGAGTTGCAG
Acetobacter-selective primer	CCCTTATGTCCTGGGCTACA	TCACCGGCTTAAGGTCAAAC
Universal primer (27F, 1492R)	AGAGTTTGATCMTGGCTCAG	TACGGYTACCTTGTTACGACTT
