# Supplementary material for: The role of commensal microbes in the lifespan of Drosophila melanogaster
Source: Aging (Albany NY). 2019 Jul 12;11(13):4611–40. doi: 10.18632/aging.102073 (PMC6660043; doi:10.18632/aging.102073)
Supplement: Supplementary Table 3 [file aging-11-102073-s002.docx]

**Supplementary Table 3. Bacteria listed by 454-Pyrosequencing.**

| **Taxonomy** | **Phylum** | **Species** | **Y**^¶^**#1** | **Y #2** | **Y #3** | **O**^§^**#1** | **O #2** | **O #3** | **SUM (Ratio)** |
| --- | --- | --- | --- | --- | --- | --- | --- | --- | --- |
| *Bacteria;;;Proteobacteria;;Alphaproteobacteria;;Rhodospirillales;;Acetobacteraceae;;Acetobacter;Acetobacter malorum* | *Proteobacteria* | *Acetobacter malorum* | 0.08 | 0.04 | 3.12 | 44.71 | 66.01 | 52.80 | 166.75 |
| *Bacteria;;;Proteobacteria;;Alphaproteobacteria;;Rhodospirillales;;Acetobacteraceae;;Acetobacter;Acetobacter persici* | *Proteobacteria* | *Acetobacter persici* | 81.38 | 17.47 | 28.02 | 12.44 | 4.54 | 0.78 | 144.64 |
| *Bacteria;;;Firmicutes;;Bacilli;;Lactobacillales;;Lactobacillaceae;;Lactobacillus;Lactobacillus plantarum* group | *Firmicutes* | *Lactobacillus plantarum* group | 16.58 | 0.77 | 4.61 | 30.33 | 22.26 | 20.81 | 95.36 |
| *Bacteria;;;Firmicutes;;Bacilli;;Lactobacillales;;Lactobacillaceae;;Lactobacillus;Lactobacillus brevis* | *Firmicutes* | *Lactobacillus brevis* | 0.00 | 45.79 | 56.96 | 0.03 | 3.71 | 0.25 | 106.74 |
| *Bacteria;;;Firmicutes;;Bacilli;;Lactobacillales;;Leuconostocaceae;;Leuconostoc;Leuconostoc pseudomesenteroides* | *Firmicutes* | *Leuconostoc pseudomesenteroides* | 0.00 | 0.00 | 0.00 | 8.01 | 1.48 | 24.50 | 33.99 |
| *Bacteria;;;Proteobacteria;;Alphaproteobacteria;;Rhodospirillales;;Acetobacteraceae;;Komagataeibacter;Komagataeibacter medellinensis* | *Proteobacteria* | *Komagataeibacter medellinensis* | 0.67 | 34.20 | 6.30 | 0.61 | 0.03 | 0.00 | 41.82 |
| *Bacteria;;;Firmicutes;;Bacilli;;Lactobacillales;;Lactobacillaceae;;Lactobacillus;Lactobacillus xiangfangensis* | *Firmicutes* | *Lactobacillus xiangfangensis* | 0.37 | 0.05 | 0.14 | 0.82 | 0.36 | 0.28 | 2.02 |
| *Bacteria;;;Proteobacteria;;Alphaproteobacteria;;Rhodospirillales;;Acetobacteraceae;;Acetobacter;Acetobacter*_uc | *Proteobacteria* | *Acetobacter*_uc | 0.04 | 0.04 | 0.12 | 1.01 | 0.51 | 0.10 | 1.81 |
| *Bacteria;;;Proteobacteria;;Alphaproteobacteria;;Rhodospirillales;;Acetobacteraceae;;Acetobacter;Acetobacter orleanensis* | *Proteobacteria* | *Acetobacter orleanensis* | 0.04 | 0.00 | 0.00 | 0.93 | 0.26 | 0.02 | 1.26 |
| *Bacteria;;;Firmicutes;;Bacilli;;Lactobacillales;;Lactobacillaceae;;Lactobacillaceae*_uc*;Lactobacillaceae_*uc_s | *Firmicutes* | *Lactobacillaceae*_  uc_s | 0.08 | 0.07 | 0.10 | 0.27 | 0.15 | 0.17 | 0.84 |
| *Bacteria;;;Proteobacteria;;Alphaproteobacteria;;Rhodospirillales;;Acetobacteraceae;;Acetobacter;Acetobacter estunensis* | *Proteobacteria* | *Acetobacter estunensis* | 0.10 | 0.75 | 0.16 | 0.00 | 0.00 | 0.00 | 1.00 |
| *Bacteria;;;Firmicutes;;Bacilli;;Lactobacillales;;Lactobacillales_*uc*;;Lactobacillales_*uc_g*;Lactobacillales_*uc_s | *Firmicutes* | *Lactobacillales*_uc_s | 0.06 | 0.04 | 0.02 | 0.05 | 0.32 | 0.06 | 0.55 |
| *Bacteria;;;Proteobacteria;;Alphaproteobacteria;;Rhodospirillales;;Acetobacteraceae;;Acetobacter;Acetobacter aceti* | *Proteobacteria* | *Acetobacter aceti* | 0.27 | 0.22 | 0.12 | 0.03 | 0.00 | 0.00 | 0.64 |
| *Bacteria;;;Firmicutes;;Bacilli;;Lactobacillales;;Lactobacillaceae;;Lactobacillus;Lactobacillus*_  uc | *Firmicutes* | *Lactobacillus*_uc | 0.02 | 0.07 | 0.04 | 0.25 | 0.08 | 0.00 | 0.46 |
| *Bacteria;;;Proteobacteria;;Alphaproteobacteria;;Rhodospirillales;;Acetobacteraceae;;Acetobacteraceae*_uc*;Acetobacteraceae*_uc_s | *Proteobacteria* | *Acetobacteraceae*_  uc_s | 0.06 | 0.05 | 0.02 | 0.12 | 0.11 | 0.04 | 0.40 |
| *Bacteria;;;Firmicutes;;Bacilli;;Bacilli_*uc*;;Bacilli*_uc_f;*;Bacilli_uc_g;Bacilli*_uc_s | *Firmicutes* | *Bacilli*_uc_s | 0.02 | 0.00 | 0.06 | 0.05 | 0.02 | 0.00 | 0.15 |
| *Bacteria;;;Firmicutes;;Bacilli;;Lactobacillales;;Leuconostocaceae;;Leuconostoc;Leuconostoc*_uc | *Firmicutes* | *Leuconostoc*_uc | 0.00 | 0.00 | 0.00 | 0.04 | 0.02 | 0.05 | 0.11 |
| *Bacteria;;;Proteobacteria;;Alphaproteobacteria;;Rhodospirillales;;Rhodospirillales*_uc*;;Rhodospirillales*_uc_g*;Rhodospirillales*_uc_s | *Proteobacteria* | *Rhodospirillales*_  uc_s | 0.02 | 0.04 | 0.00 | 0.04 | 0.01 | 0.01 | 0.12 |
| *Bacteria;;;Proteobacteria;;Alphaproteobacteria;;Rhodospirillales;;Acetobacteraceae;;Komagataeibacter;Komagataeibacter kombuchae* | *Proteobacteria* | *Komagataeibacter kombuchae* | 0.00 | 0.04 | 0.00 | 0.06 | 0.00 | 0.00 | 0.10 |
| *Bacteria;;;Firmicutes;;Bacilli;;Lactobacillales;;Leuconostocaceae;;Leuconostocaceae*_uc*;Leuconostocaceae*_uc_s | *Firmicutes* | *Leuconostocaceae*_  uc_s | 0.00 | 0.00 | 0.00 | 0.00 | 0.00 | 0.08 | 0.08 |
| *Bacteria;;;Proteobacteria;;Alphaproteobacteria;;Rhodospirillales;;Acetobacteraceae;;Komagataeibacter;Komagataeibacter europaeus* group | *Proteobacteria* | *Komagataeibacter europaeus* group | 0.00 | 0.09 | 0.00 | 0.00 | 0.00 | 0.00 | 0.09 |
| *Bacteria;;;Bacteria*_uc*;;Bacteria*_uc_c*;;Bacteria_*uc_o*;;Bacteria_uc_f;;Bacteria_uc_g;Bacteria*_uc_s | *Bacteria*_uc | *Bacteria*_uc_s | 0.02 | 0.00 | 0.00 | 0.04 | 0.00 | 0.01 | 0.07 |
| *Bacteria;;;Proteobacteria;;Alphaproteobacteria;;Rhodospirillales;;Acetobacteraceae;;Acetobacter;Acetobacter cibinongensis* | *Proteobacteria* | *Acetobacter cibinongensis* | 0.00 | 0.00 | 0.00 | 0.05 | 0.00 | 0.00 | 0.05 |
| *Bacteria;;;Cyanobacteria;;*JF737898_c;;JF417809_o*;;*JF417809_f*;;*AJ583204_g*;*AJ583204_s | *Cyanobacteria* | AJ583204_s | 0.00 | 0.05 | 0.02 | 0.00 | 0.00 | 0.00 | 0.07 |
| *Bacteria;;;Proteobacteria;;Alphaproteobacteria;;Rhodospirillales;;Acetobacteraceae;;Komagataeibacter;Komagataeibacter kakiaceti* | *Proteobacteria* | *Komagataeibacter kakiaceti* | 0.00 | 0.04 | 0.02 | 0.01 | 0.00 | 0.00 | 0.07 |
| *Bacteria;;;Proteobacteria;;Alphaproteobacteria;;Alphaproteobacteria_uc;;Alphaproteobacteria_uc_f;;Alphaproteobacteria_uc_g;Alphaproteobacteria*_uc_s | *Proteobacteria* | *Alphaproteobacteria*_  uc_s | 0.00 | 0.02 | 0.00 | 0.03 | 0.00 | 0.00 | 0.04 |
| *Bacteria;;;Proteobacteria;;Betaproteobacteria;;Burkholderiales;;Ralstonia_f;;Ralstonia;*AM713401_s | *Proteobacteria* | AM713401_s | 0.04 | 0.02 | 0.00 | 0.00 | 0.00 | 0.00 | 0.06 |
| *Bacteria;;;Proteobacteria;;Alphaproteobacteria;;Rhodospirillales;;Acetobacteraceae;;Acetobacter;Acetobacter indonesiensis* | *Proteobacteria* | *Acetobacter indonesiensis* | 0.00 | 0.04 | 0.00 | 0.01 | 0.00 | 0.00 | 0.05 |
| *Bacteria;;;Proteobacteria;;Proteobacteria_uc;;Proteobacteria_uc_o;;Proteobacteria_uc_f;;Proteobacteria_uc_g;Proteobacteria*_uc_s | *Proteobacteria* | *Proteobacteria*_uc_s | 0.02 | 0.00 | 0.02 | 0.01 | 0.00 | 0.00 | 0.05 |
| *Bacteria;;;Firmicutes;;Bacilli;;Bacillales;;Sporolactobacillaceae;;Tuberibacillus;Tuberibacillus*_uc | *Firmicutes* | *Tuberibacillus_*uc | 0.00 | 0.00 | 0.00 | 0.00 | 0.00 | 0.02 | 0.02 |
| *Bacteria;;;Proteobacteria;;Betaproteobacteria;;Burkholderiales;;Oxalobacteraceae;;Massilia;Massilia varians* | *Proteobacteria* | *Massilia varians* | 0.00 | 0.00 | 0.04 | 0.00 | 0.00 | 0.00 | 0.04 |
| *Bacteria;;;Firmicutes;;Bacilli;;Bacillales;;Bacillaceae;;Geobacillus;Geobacillus stearothermophilus* | *Firmicutes* | *Geobacillus stearothermophilus* | 0.00 | 0.00 | 0.04 | 0.00 | 0.00 | 0.00 | 0.04 |
| *Bacteria;;;Firmicutes;;Firmicutes_uc;;Firmicutes_uc_o;;Firmicutes_uc_f;;Firmicutes_uc_g;Firmicutes*_uc_s | *Firmicutes* | *Firmicutes*_uc_s | 0.00 | 0.00 | 0.00 | 0.01 | 0.00 | 0.01 | 0.03 |
| *Bacteria;;;Acidobacteria;;Solibacteres;;EU445199_o;;EU445199_f;;EU445199_g;JX001319*_s | *Acidobacteria* | JX001319_s | 0.00 | 0.04 | 0.00 | 0.00 | 0.00 | 0.00 | 0.04 |
| *Bacteria;;;Proteobacteria;;Betaproteobacteria;;Burkholderiales;;Ralstonia_f;;Ralstonia;Ralstonia pickettii* | *Proteobacteria* | *Ralstonia pickettii* | 0.04 | 0.00 | 0.00 | 0.00 | 0.00 | 0.00 | 0.04 |
| *Bacteria;;;Firmicutes;;Bacilli;;Bacillales;;Bacillaceae;;Geobacillus;Geobacillus*_uc | *Firmicutes* | *Geobacillus*_uc | 0.00 | 0.00 | 0.00 | 0.00 | 0.02 | 0.00 | 0.02 |
| *Bacteria;;;Planctomycetes;;*HQ681992_c*;;*HQ681992_o*;;*HQ681992_f*;;*HQ681992_f_uc*;*HQ681992_f_uc_s | *Planctomycetes* | HQ681992_f_uc_s | 0.04 | 0.00 | 0.00 | 0.00 | 0.00 | 0.00 | 0.04 |
| *Bacteria;;;Proteobacteria;;Gammaproteobacteria;;DQ906757_o;;DQ906757_f;;DQ906757_g;DQ906757_g_uc* | *Proteobacteria* | DQ906757_g_uc | 0.00 | 0.00 | 0.00 | 0.00 | 0.01 | 0.00 | 0.01 |
| *Bacteria;;;Proteobacteria;;Betaproteobacteria;;Burkholderiales;;Sphaerotilus_f;;Aquabacterium;Aquabacterium commune* | *Proteobacteria* | *Aquabacterium commune* | 0.00 | 0.00 | 0.00 | 0.01 | 0.00 | 0.00 | 0.01 |
| *Bacteria;;;Proteobacteria;;Alphaproteobacteria;;Rhodospirillales;;Acetobacteraceae;;Acetobacter;Acetobacter nitrogenifigens* | *Proteobacteria* | *Acetobacter nitrogenifigens* | 0.02 | 0.00 | 0.00 | 0.00 | 0.00 | 0.00 | 0.02 |
| *Bacteria;;;Proteobacteria;;Alphaproteobacteria;;Rhodospirillales;;Acetobacteraceae;;Komagataeibacter;Komagataeibacter hansenii* | *Proteobacteria* | *Komagataeibacter hansenii* | 0.00 | 0.02 | 0.00 | 0.00 | 0.00 | 0.00 | 0.02 |
| *Bacteria;;;Proteobacteria;;Alphaproteobacteria;;Sphingomonadales;;Sphingomonadaceae;;Sphingomonas;Sphingomonas aquatilis* group | *Proteobacteria* | *Sphingomonas aquatilis* group | 0.00 | 0.00 | 0.02 | 0.00 | 0.00 | 0.00 | 0.02 |
| *Bacteria;;;Proteobacteria;;Alphaproteobacteria;;Caulobacterales;;Caulobacteraceae;;Brevundimonas;Brevundimonas bullata* | *Proteobacteria* | *Brevundimonas bullata* | 0.02 | 0.00 | 0.00 | 0.00 | 0.00 | 0.00 | 0.02 |
| *Bacteria;;;Proteobacteria;;Alphaproteobacteria;;*AY957891_o*;;*AY957891_f*;;*AY957891_g*;*AY957891_s | *Proteobacteria* | AY957891_s | 0.00 | 0.00 | 0.02 | 0.00 | 0.00 | 0.00 | 0.02 |
| *Bacteria;;;Proteobacteria;;Alphaproteobacteria;;Rhizobiales;;Bradyrhizobiaceae;;Bradyrhizobium;Bradyrhizobium japonicum* group | *Proteobacteria* | *Bradyrhizobium japonicum* group | 0.02 | 0.00 | 0.00 | 0.00 | 0.00 | 0.00 | 0.02 |
| *Bacteria;;;Actinobacteria;;Actinobacteria_c;;Micrococcales;;Micrococcaceae;;Arthrobacter;Arthrobacter histidinolovorans* group | *Actinobacteria* | *Arthrobacter histidinolovorans* group | 0.00 | 0.02 | 0.00 | 0.00 | 0.00 | 0.00 | 0.02 |
| *Bacteria;;;Proteobacteria;;Alphaproteobacteria;;Rhodospirillales;;Acetobacteraceae;;Komagataeibacter;Komagataeibacter*_uc | *Proteobacteria* | *Komagataeibacter*_  uc | 0.00 | 0.02 | 0.00 | 0.00 | 0.00 | 0.00 | 0.02 |
| *Bacteria;;;Firmicutes;;Bacilli;;Lactobacillales;;Lactobacillaceae;;Lactobacillus;Lactobacillus yonginensis* | *Firmicutes* | *Lactobacillus yonginensis* | 0.00 | 0.00 | 0.00 | 0.00 | 0.01 | 0.00 | 0.01 |
| *Bacteria;;;Firmicutes;;Bacilli;;Bacillales;;Bacillaceae;;Bacillus;Bacillus aryabhattai* | *Firmicutes* | *Bacillus aryabhattai* | 0.00 | 0.00 | 0.02 | 0.00 | 0.00 | 0.00 | 0.02 |
| *Bacteria;;;Proteobacteria;;Alphaproteobacteria;;Rhodospirillales;;Acetobacteraceae;;Komagataeibacter;Komagataeibacter maltaceti* group | *Proteobacteria* | *Komagataeibacter maltaceti* group | 0.00 | 0.02 | 0.00 | 0.00 | 0.00 | 0.00 | 0.02 |
| *Bacteria;;;Proteobacteria;;Alphaproteobacteria;;Rhizobiales;;Methylobacteriaceae;;Methylobacterium;Methylobacterium jeotgali* | *Proteobacteria* | *Methylobacterium jeotgali* | 0.00 | 0.00 | 0.02 | 0.00 | 0.00 | 0.00 | 0.02 |

^¶^ Y indicates Young; ^§^ O indicates Old
