# Supplementary material for: The role of commensal microbes in the lifespan of Drosophila melanogaster
Source: Aging (Albany NY). 2019 Jul 12;11(13):4611–40. doi: 10.18632/aging.102073 (PMC6660043; doi:10.18632/aging.102073)
Supplement: Supplementary Table 12 [file aging-11-102073-s001.docx]

**Supplementary Table 12. Summary of axenic fly lifespan data in previous reports.**

| **No.** | **Author** | **Strain** | **Sex** | **Food contents** | **Dominant bacteria** | **Axenic method** | | **Lifespan change** | **Reference** |
| --- | --- | --- | --- | --- | --- | --- | --- | --- | --- |
|  |  |  |  |  |  | **Bleaching** | **Antibiotics** |  |  |
| 1 | Lee *et al*. | *w^1118^* | Female | 2.5% yeast  11% sugar  5.2% cornmeal  1% agar  0.04% methyl 4 hydroxybenzoate  0.5% propionic acid | *Acetobacter persici* JCM25330(T)  *Acetobacter malorum* LMG1746(T)  *Lactobacillus brevis* ATCC14869(T)  *Lactobacillus plantarum* ATCC14917(T) | 3 % sodium hypochlorite | 640 μg/ml doxycycline 640 μg/ml ampicillin 1 mg/ml kanamycin | Increased | Our study |
| 2 | Brummel *et al*. | *w^1118^* | Male | 5% dextrose 2.5% sucrose 1.5% yeast 17% cornmeal 0.9% agar 0.09% propionic acid 0.09% phosphoric acid | *Lactobacillus Gluconobacter Enterobacter Anaerococcus* | 2.7 % sodium hypochlorite | 500 μg/ml ampicillin 50 μg/ml tetracycline 200 μg/ml rifamycin | Bleaching: 30% decreased (22 °C) Antibiotics: 35% decreased (25 °C) | Brummel *et al*. (2004) *PNAS* 101(35): 12974-12979 |
| 3 | Ren *et al*. | Canton-S and  Oregon-R | Male | 10.5% dextrose 0.75% Agar 2.6% yeast 5 % cornmeal Tegosept Propionic acid | *Acetobacter aceti Acetoacter tropicalis Acetobacter pasterianus Lactobacilles plantarum Lactobacillus sp.* MR-2 *Cladosporium sphaerospermum Lactobacillus homohiochii Lactobacillus fructivorans Acetobacter tropicalis Lactobacillus brevis* | 0.25% Clorox and  0.04% n-alkyl dimethyl benzyl ammonium chloride | 640 μg/ml doxycycline 640 μg/ml ampicillin 1 mg/ml kanamycin | Bleaching: increased  (n.s.^†^) Antibiotics: No increased  (n.s.^†^) | Ren *et al*. (2007) *Cell Metab.* 6(2): 144-152 |
| 4 | Ridley *et al*. | Canton-S | Male | 9.6% glucose 4.8% inactive dry yeast 1.4% agar | including *Acetobacter pomorum* strain EW816 and *Lactobacillus plantarum* strain WCFS1 | 10% sodium hypochlorite solution |  | Increased  (n.s.^†^) | Ridley *et al*. (2012) *PLoS One* 7(5): e36765 |
| 5 | Lee *et al*. | *w^1118^* | Male | Standard cornmeal-agar medium |  | First generation, 2.7 % sodium hypochlorite |  | Increased | Lee *et al*. (2013) *Cell* 153(4): 797-811 |
| 6 | Petkau *et al*. | *w^1118^* | Male and female | Cornmeal-dextrose-yeast media |  |  | 100 g/ml ampicillin  50 g/ml vancomycin  100 g/ml neomycin  100 g/ml metronidazole | Increased | Petkau *et al*. (2014) *J Biol Chem.* 289(41): 28719 -28729 |
| 7 | Clark *et al*. | *w^1118^*  and  Canton-S | Female | Standard cornmeal medium 1% agar 3% brewer’s yeast  1.9% sucrose 3.8% dextrose 9.1% cornmeal |  | 3% sodium hypochlorite |  | Increased | Clark *et al*. (2015) *Cell Rep.* 12(10): 1656-1667 |
| 8 | Yamada *et al*. | Wolbachia–free Dahomey | Male | 0.5% yeast extract  5% sucrose  8.6% cornmeal  0.5% agar  0.4% propionic acid  0.035% phosphoric acid | *Issatchenkia orientalis*  *Lactobacillus plantarum*  *Acetobacter indonesiensis*  *Saccharomyces cerevisiae* | 3% sodium hypochlorite |  | No effect | Yamada *et al*. (2015) *Cell Rep.* 10(6): 865–872 |
| 9 | Galenza *et al*. | *w^1118^* | Male and female | 10 % D-glucose 5 % starch 7 % casein 5 % palmitic acid 1 % ethanol | *Acetobacter Lactobacillus Streptococcus Lactococcus Staphylococcus Ralstonia Stenotrophomonas Methylobacterium Propionobacterium Bradyrhizobium Herbaspirillum Micrococcus Finegoldia Abiotrophia Neisseria* |  | 100 μg/ml ampicillin 50 μg/ml vancomycin 100 μg/ml neomycin 100 μg/ml metronidazole | Increased | Galenza *et al*. (2016) *Biol Open.* 5: 165-173 |
| 10 | Fast *et al*. | *w^1118^* | Virgin female | Autoclaved standard cornmeal medium |  | 10% sodium hypochlorite solution | 100 μg/ml ampicillin 100 μg/ml metronidazole 40 μg/ml vancomycin 100 μg/ml neomycin | Antibiotics  (increased and no effect)  Bleach  (no effect) | Fast *et al*. (2016) *BioRxiv* doi.org/10.1101/049981 |
| 11 | Li *et al*. | *w^1118^* | Female | Yeast/molasses-based standard fly food  1.38% agar 2.2% molasses 7.5 % malt extract 1.8 % dry yeast 8 % corn flour 1 % soy flour 0.625 % propionic acid 0.2% Methyl 4-Hydroxybenzoate in 0.72% ethanol | *Acetobacter pasteurianus Escherichia albertii Lactobacillus fructivorans Lactobacillus homohiochii Serratia entomophila Lactobacillus senmaizukei Stenotrophomonas pavanii Methylobacterium jeotgali* |  | 50 μg/ml ampicillin 50 μg/ml tetracycline 50 μg/ml erythromycin 50 μg/ml kanamycin | No effect (29 °C) | Li *et al*. (2016) *Cell Host Microbe* 19(2): 240-253 |
| 12 | Dantoft *et al*. | Oregon-R | Male and female mixed | 1.26% dry yeast,  0.05 % syrup,  4 % instant mashed potato powder,  1 % agar 0.85% Nipagin ®  and 0.025 % ascorbic acid | *Actinobacteria Alphaproteobacteria/other Alphaproteobacteria/Rhodospirillales Bacteria/other Firmicutes/Lactobacillales Firmicutes/other Gammaproteobacteria/other Gammaproteobacteria/Pasteurellales Proteobacteria/other Unclassified* |  | 100 μg/ml of carbenicillin 100 μg/ml of neomycin 50 μg/ml of vancomycin  100 μg/ml of metronidazole | 25% increased | Dantoft *et al*. (2016) *J Innate Immun.* 8: 412-426 |
| 13 | Gould *et al*. | Wolbachia–free  Canton-S | Female | 6.67% cornmeal 2.7% active dry yeast 1.6% sucrose 0.75% sodium tartrate 0.73% ethanol 0.68% agar 0.46% propionic acid 0.09% methylparaben 0.06% calcium chloride 0.01% molasses |  | Multiple generations, 0.6% sodium hypochlorite |  | Increased | Gould *et al*. (2017) *BioRxiv* doi.org/10.1101/232959 |
| 14 | Téfit and Leulier | *yw* | Male and female | 5% inactivated yeast  8% cornmeal  1% agar  0.52% methylparaben sodium salt  0.4 ml 99% propionic acid |  | Bleaching | 50 μg/L ampicillin  50 μg/L kanamycin  50 μg/L tetracycline  15 μg/L erythromycin | Increased | Téfit and Leulier (2017) *J Exp Biol.* 220: 900-907 |
| 15 | Loch *et al*. | *Tub^GS^* or *Ti^GS^* /UAS-*Dro* or *CecA1* | Female | <Rearing> 5.7 % cornmeal 1.15 % yeast 0.6 % agar-agar 7% sugar beet molasses 1.14 % Nipagin <Experiment> 7.5% yeast autolysate  7.5% glucose 2.1% ethanol 2% Kobe I agar  0.3% Nipagin | *Lactobacillus* (60.87%) *Acetobacter* (38.96%) *Pseudomonas* (0.07%) *Uncultured bacteria* (0.04%) *Erwinia* (0.02%) *Propionibacterium* (0.02%) *Cupriabidus* (<0.01%) *Escherichia* (<0.01%) |  | 500 μg/ml ampicillin  50 μg/ml tetracycline  200 μg/ml rifampicin | Tub^GS^>Dro (RU-) 15.6% increased  Ti^GS2^>Dro (RU-) 11.5% increased | Loch *et al*. (2017) *PLoS One* 12(5): e0176689 |
| 16 | Obata *et al*. | *w^iso31^* | Male and female | 2.34% autolysed yeast extract 5.85% glucose 6.63% cornmeal 0.702% agar 1.95% antimycotic solution containing 0.04% bavistan and 10% nipagin | *Acetobacter aceti Acetobacter sicerae Acetobacter orleanensis Acetobacter pomorum Acetobacter pasteurianus Lactobacillus plantarum  Lactobacillus pentosus* |  | RTA: 200 μg/mL rifamycin 50 μg/mL tetracycline 500 μg/mL ampicillin  MVNTA: 100 μg/mL metronidazole 50 μg/mL vancomycin 100 μg/mL neomycin 50 μg/mL tetracycline 100 μg/mL ampicillin | RTA 21.8% increased MVNTA 18.2% increased | Obata *et al*. (2018) *Nat Commun.* 9(975): 1-12 |
| 17 | Sannino *et al*. | Wolbachia–free  Canton-S | Male and female | 5% yeast 4% sucrose 6% cornmeal | *Acetobacter pomorum* DmCS_004 *Acetobacter tropicalis* DmCS_006 *Lactobacillus brevis* DmCS_003 *Lactobacillus plantarum* DmCS_001 *Acetobacter pasteurianus* SKU1108 | 0.6% hypochlorite |  | No effect | Sannino *et al*. (2018) *mBio* 9(2): e00155-18 |
| 18 | Iatsenko *et al*. | *w^1118^* | Male | 0.62% agar  5.88% cornmeal  5.88% inactivated dried yeast  2.67 ml of a 10% solution of methyl- paraben in 85% ethanol  6 ml fruit juice  0.48 ml 99% propionic acid | *Lactobacillus plantarum* SD | 3% sodium hypochlorite |  | Increased | Iatsenko *et al*. (2018) *Immunity* 49(5), 929-942 |

^†^ n.s. Statistically non-significance
